# Sustained-Release Spermidine Hydrogel Inhibits M1 Macrophage Polarization and Promotes Tissue Repair for Spinal Cord Injury Repair

**DOI:** 10.34133/bmr.0247

**Published:** 2025-09-11

**Authors:** Yongjun Luo, Xiao Zhang, Qian Luo, Liang Wu, Shubo Gu, Zuozhi Xie, Xiaolin Zeng, Yili Xu, Yao Wu, Hao Zhou, Tao Xu, Zheng Zhou

**Affiliations:** ^1^ Emergency and Critical Care Medicine Department, The First Affiliated Hospital with Nanjing Medical University, Nanjing, China.; ^2^ Department of Orthopedics, The Fourth Affiliated Hospital of Soochow University, Suzhou, Jiangsu, China.; ^3^ Division of Spine Surgery, Department of Orthopedic Surgery, Nanjing Drum Tower Hospital, Affiliated Hospital of Medical School, Nanjing University, Nanjing, China.; ^4^ Department of Orthopedics, The Second Affiliated Hospital of Soochow University, Suzhou, China.; ^5^ Department of Oncology, The Second Affiliated Hospital of Nanjing Medical University, Nanjing, Jiangsu, China.; ^6^ Key Laboratory of Emergency and Trauma of Ministry of Education, Hainan Medical University, Haikou, China.

## Abstract

The use of injectable hydrogels represents a viable approach for enhancing neural repair and promoting functional restoration after spinal cord trauma. Nevertheless, the current performance of these materials is not yet optimal and further optimization is necessary. Engineering a cell-free hydrogel delivery system with sustained anti-inflammatory capacity is of great relevance for advancing therapeutic strategies in spinal cord injury (SCI). Here, we fabricated a biomimetic hydrogel incorporating spermidine to modulate the post-injury immune microenvironment. The material was constructed by photocrosslinking aldehyde-modified methacrylated hyaluronic acid (AHAMA) through dynamic Schiff base chemistry, enabling controlled and prolonged spermidine release. This hydrogel demonstrated expedited gelation kinetics coupled with stable and exceptional mechanical properties. In addition, the cell-free AHAMA hydrogels have substantially enhanced the cellular–matrix interactions and facilitated neuronal integration. Furthermore, the spermidine-loaded hydrogel exerted potent immunomodulatory effects by suppressing M1 macrophage (classically activated macrophage) polarization through activation of STAT1 (signal transducer and activator of transcription 1) signaling axis. In vivo assessments demonstrated enhanced neuroregeneration and axonal elongation at the lesion site, which translated into marked improvements in locomotor function in the murine SCI model. Collectively, the combination of sustained spermidine release with a bioinspired, cell-free AHAMA hydrogel scaffold offers an effective therapeutic approach to modulate inflammation and enhance tissue repair in the injured spinal cord environment.

## Introduction

As a severe and intricately complex neurological disorder, spinal cord injury (SCI) leads to disturbances in sensory integration and motor output below the lesion site, posing marked challenges in the field of spinal surgery. Ultimately, this leads to grave and potentially fatal complications, inflicting enormous societal health burdens and significant economic losses [1]. Upon SCI, a cascade of pathological responses ensues at the site of the lesion, including neuronal loss, oxidative stress, inflammation, and formation of glial scars. These processes give rise to chemical and physical barriers that impede the spontaneous regeneration and repair of damaged nerves, constraining the natural ability to recover [2]. The mammalian central nervous system (CNS) possesses inherently limited regenerative potential, and there are restricted clinical treatment approaches, leading to notable limitations in the treatment of SCI [3]. Recent progress in biomaterials science has substantially broadened the therapeutic prospects for addressing SCI [4].

Naturally derived polymeric materials are increasingly employed in biomedical engineering owing to their inherent low immunogenicity and high tissue affinity [5]. Among them, hyaluronic acid (HA), an essential polysaccharide abundantly present in the extracellular matrix (ECM), contributes considerably to neurodevelopmental processes within the CNS [6]. Its extended linear chain structure facilitates moisture retention and imparts viscoelastic properties to the scaffold environment [6]. High-molecular-weight HA has been reported to attenuate astrocytic expansion and suppress the accumulation of chondroitin sulfate proteoglycans (CSPGs), thereby limiting microglial infiltration and neuroinflammatory injury, ultimately contributing to the restoration of neural function [7].

However, several intrinsic drawbacks of HA still hinder its broader biomedical implementation, such as limited structural integrity, suboptimal cellular anchorage, and rapid enzymatic breakdown in physiological environments [8]. To address these challenges, chemical bond modification of injectable adhesive HA hydrogels has emerged as crucial strategies to enhance their applicability [6]. The injectable hydrogel, being in a fluid state, offers the advantage of being suitable for minimally invasive surgery and facile delivery to injured tissues, making it highly applicable for in situ treatment of injuries [9]. Importantly, this injectable hydrogel can be precisely tuned to recapitulate physicomechanical and biochemical context of the ECM framework, thereby creating a permissive environment for neural repair within the injured spinal cord [10]. Therefore, aldehyde-modified methacrylated hyaluronic acid (AHAMA) hydrogels represent a promising bioengineering material for facilitating neural regeneration following spinal trauma.

Polyamines are small, positively charged molecules synthesized via the metabolic conversion of L-arginine [11]. Recognized for their intrinsic anti-inflammatory activity, polyamines have garnered significant attention in recent studies [12]. Spermidine, a natural component derived from the polyamine family, was initially isolated from semen and is widely present in eukaryotic organisms. Additionally, spermidine exhibits the capability to interact with polyanions such as nucleic acids, proteins, and adenosine triphosphate (ATP), thus playing a role in preserving DNA genomic stability and modulating gene transcription and translation. It plays crucial roles in cellular processes including autophagy, apoptosis, oxidative stress response, angiogenesis, and intercellular communication [13]. Moreover, spermidine is critically involved in the regulation of cellular proliferation and division [14]. Therefore, spermidine exhibits excellent biocompatibility and distinct physicochemical characteristics, which render it highly suitable for the surface modification of diverse biomaterials in biomedical applications [15].

Recent studies have demonstrated that exogenous spermidine supplementation alleviates neurological dysfunction and inflammatory responses in experimental autoimmune encephalomyelitis models [16], preserves dopaminergic neuronal integrity in Parkinsonian rats [17], and exerts both neuroprotective and anti-inflammatory effects in aging-related neurodegeneration [18]. Furthermore, excessive inflammatory response in SCI is a crucial factor influencing the prognosis [19]. Macrophages, as important inflammatory cells in SCI, polarize into M1 phenotype during pathological progression, exerting pro-inflammatory effects and damaging injured cells [20]. Macrophage polarization toward the M1 phenotype is predominantly driven by signal transducer and activator of transcription 1 (STAT1) signaling, which subsequently induces elevated output of inflammation-promoting mediators such as iNOS (inducible nitric oxide synthase) [21]. Given the potential anti-inflammatory role of spermidine within the nervous system, elucidating its regulatory effects on M1 macrophage polarization is of considerable importance for enhancing post-injury repair processes in spinal cord pathology. Therefore, based on the promising application prospects of spermidine in biomaterials and its unique biological effects in the nervous system, the development of spermidine-releasing biomaterials for the treatment of SCI holds great potential. However, the therapeutic deployment of spermidine-releasing scaffolds in SCI repair has been minimally explored.

Here, we engineered an AHAMA hydrogel, which forms imine bonds through a dynamic Schiff base reaction with spermidine, enabling controlled release of spermidine. This hydrogel exhibited rapid gelation and stable mechanical properties. Furthermore, spermidine released in a controlled manner inhibited M1 macrophage polarization. In vivo assessments further confirmed hydrogel-induced neurogenesis and axonal remodeling at the lesion site, which translated into marked improvements in locomotor performance in the murine SCI model. In summary, AHAMA hydrogels combined with sustained release of therapeutic spermidine suppressed M1 macrophage polarization, resulting in the effective repair of SCI.

## Materials and Methods

### Synthesis process

#### Chemical preparation of aldehyde-modified hyaluronic acid

Aldehyde-modified hyaluronic acid (AHA) was obtained using a standard protocol as outlined in earlier studies [22]. In brief, HA (1.0 g) was dispersed in 100 ml of deionized water until fully solubilized, after which 0.5 g of sodium periodate was introduced slowly under continuous agitation. The reaction mixture was quenched by adding 1 ml of ethylene glycol to eliminate residual sodium periodate. After 1 h of incubation, the resulting solution was subjected to dialysis against deionized water, followed by freeze-drying to obtain the final product. The proton (^1^H) NMR spectrum of the synthesized AHA was acquired on a 400-MHz Bruker spectrometer (Bruker, Germany) after dissolution in D₂O.

#### Synthesis of AHAMA

A reported synthetic approach was adopted for the preparation of AHAMA [22]. Initially, AHA (1 g) was dispersed thoroughly in 100 ml of Milli-Q water, followed by the gradual addition of 1 ml of methacrylate (MA) under agitation. pH was brought to a range of 8.0 to 8.5 by titration with 1 M NaOH, and the reaction mixture was maintained at 4 °C under constant mixing for 24 h. Thereafter, the solution was dialyzed against ddH₂O, 0.1 M NaCl solution, and 25% (v/v) ethanol for 48 h and lyophilized. The ^1^H NMR spectrum of AHAHA was obtained using a Bruker 400-MHz NMR spectrometer (Bruker, Germany) after dissolution in D₂O.

#### Synthesis of spermidine-conjugated AHAMA

Spermidine-conjugated AHAMA (SpdHAMA) was synthesized through dynamic Schiff base formation involving the amino groups of spermidine and the aldehyde functionalities on AHAMA. One gram of AHAMA was dispersed in 100 ml of ultrapure water and stirred until complete solubilization, after which spermidine was introduced. To ensure full conversion, the system was agitated steadily at room temperature for 72 h. The resulting solution underwent dialysis against deionized water for 48 h, followed by lyophilization to yield SpdHAMA. All samples were named L-SpdHAMA (low-spermidine-modified AHAMA), M-SpdHAMA (medium-spermidine-modified AHAMA) and H-SpdHAMA (high-spermidine-modified AHAMA) according to the variation of spermidine concentration of 1, 2, and 4 mM, respectively. The ^1^H NMR spectrum of SpdHAMA was acquired using a 400-MHz NMR spectrometer (Bruker, Germany) after dissolving the sample in D₂O.

### Preparation of SpdHAMA hydrogel

To fabricate the SpdHAMA hydrogel, 5.0% (w/v) SpdHAMA and 0.3% (w/v) of the LAP (photoinitiator lithium phenyl-2,4,6-trimethylbenzoylphosphinatewere) were dissolved in phosphate-buffered saline (PBS), followed by photopolymerization under 410-nm ultraviolet (UV) irradiation (30 mW cm^−2^) for 1 min to induce gel crosslinking.

### Spectroscopic analysis using FTIR

Structural modifications of HA and its derivatives were analyzed using Fourier-transform infrared (FTIR) spectroscopy to verify functional group alterations. Freeze-dried powders of unmodified HA, AHA, AHAMA, and SpdHAMA were prepared for analysis. Infrared spectra were recorded from 4,000 to 400 cm^−1^ with data acquired at 4 cm^−1^ spectral resolution. The characteristic absorption bands of each sample was examined to confirm the incorporation of functional groups during the chemical modification process and to validate the success of the modifications.

### Gel permeation chromatography analysis

Each sample was dissolved in 1 ml of solvent, followed by standing for 5 h to ensure complete dissolution. The final solutions were filtered under aseptic conditions through a 0.22-μm filter element prior to injection. Gel permeation chromatography (GPC) measurements were conducted using a high-performance size-exclusion system (Agilent 1260 Infinity II) equipped with 2 PL-aquagel-OH columns in series (7.5 × 50 mm, 8 μm; 7.5 × 300 mm, 8 μm). A 0.1 M NaNO₃ solution served as the eluent, and the column was thermostatted at 45 °C throughout the run. The system operated at a flow rate of 1 ml per minute, with an injection volume of 20 μl. Molecular weight calibration was performed using a series of poly(ethylene glycol) reference standards.

### Degradation test and sustained releasing test in vitro

To assess in vitro degradation, 100 μl of SpdHAMA hydrogel was prepared and the initial mass (Md0) was recorded. Subsequently, the specimens were immersed within a pH 7.4 PBS bath and maintained under constant agitation preserved at low temperature (4 °C) for 28 days. At predetermined intervals (days 0, 4, 8, 12, 16, 20, 24, 28, and 32), we collected the supernatant and documented the weight of the hydrogel as Mdt. The degradation percentage (DR) was determined based on the relative mass loss using the equation: DR (%) = [(Mdt − Md0)/Md0] × 100 [23].

To obtain the release profile of the SpdHAMA hydrogel, 100 μl of the gel was incubated in 500 μl of PBS-containing medium at 37 °C. After collecting 500-μl samples, an equal volume of preheated PBS-dissolved medium was periodically replenished. For quantitative measurement of spermidine release, a Cloud-Clone ELISA kit was adopted following the manufacturer’s protocol [24]. Briefly, samples underwent centrifugation at 10,000*g* for 20 min. Subsequently, 50 μl of the supernatant and a corresponding volume of Detection Reagent A were transferred into individual wells and incubated at 37 °C for 1 h. After 3 washing steps, 100 μl of Detection Reagent B was transferred to individual wells, proceeding with an additional 30-min incubation at the same temperature. Following an additional 5 washes, each well received 90 μl of the chromogenic substrate, and an incubation phase of 10 min was applied at 37 °C to the reaction mixture. Thereafter, 50 μl of the stopping reagent was introduced to terminate the reaction. Finally, the plate was thoroughly agitated, and absorbance was immediately measured at 450 nm with a microplate reader (Thermo Fisher Scientific). The release profile was constructed using the cumulative release data obtained over time.

### In vivo measurement of spermidine levels

The skin overlying the L5-L6 (lumbar 5 to 6) region on the back was shaved and disinfected, and a 10-μl microsyringe was employed to aspirate cerebrospinal fluid (CSF) from the intrathecal space at the L5-L6 intervertebral level in mice. A quantitative analysis of spermidine in CSF was performed via HPLC-MS/MS (high-performance liquid chromatography coupled with tandem mass spectrometry) [25].

### Swelling ratio assessment

The swelling behavior of the SpdHAMA hydrogel was evaluated as follows. Firstly, the prepared hydrogel samples were lyophilized, and the dry weight of the sample was assigned as W0. Subsequently, the hydrogel samples were immersed in PBS and incubated at 37 °C for 2 h. Then, the hydrogel samples were removed from PBS and weighed as Wt at the time point of 2, 4, 8, 12, 24, and 48 h. The swelling capacity (%) was quantified by calculating the relative weight gain according to the equation: (Wt − W0)/W0 × 100%, where Wt is the swollen weight and W0 is the initial dry weight.

### Water uptake test

The in vitro water uptake of the SpdHAMA hydrogels were assessed as follows. Initially, surface moisture on the hydrogel specimens was carefully blotted using filter paper, and the wet weight was recorded as W0. Subsequently, the samples were freeze-dried and their dry weight were noted as W1. Water uptake (%) was calculated using the formula: (W0 − W1)/W0 × 100%.

### Surface morphology observation by scanning electron microscopy

Microarchitectural features of the SpdHAMA hydrogels were observed by scanning electron microscopy (SEM). The SpdHAMA hydrogels were first rinsed in PBS for 24 h. Afterward, hydrogel specimens underwent lyophilization, followed by deposition of a thin gold–palladium layer via sputtering.. Microstructural characterization was then performed using a field-emission SEM (model S3400II, Hitachi, Japan). Quantitative evaluation of pore dimensions and total void fraction in SpdHAMA hydrogels was conducted using ImageJ software based on SEM micrographs.

### Rheological analysis

Rheological measurements of SpdHAMA hydrogels were performed on a HAAKE MARS 40 rotational rheometer (Thermo Fisher Scientific, USA). A dynamic frequency sweep ranging from 0.1 to 100 Hz was conducted at 37 °C to determine the viscoelastic parameters, including storage modulus (*G*′) and loss modulus (*G*″).

A strain-dependent oscillatory test was conducted to determine the linear viscoelastic (LVE) region of the hydrogels [26]. In brief, a hydrogel solution was dispensed onto the rheometer plates, followed by the application of predetermined thermal conditions to induce gelation. Subsequently, the gel was allowed to equilibrate for an appropriate duration determined from the preceding time sweep stage, conducted without rheological monitoring.

### Compressive testing

Compressive performance of the SpdHAMA hydrogels was evaluated utilizing a standard universal testing device (Instron, High Wycombe, UK) to quantify their mechanical response under load. Graphical representation of the material’s stress–strain relationship was obtained by loading with a displacement rate of 0.1 mm per minute. The linear region of the stress–strain curve was used to calculate the compression modulus.

### Extraction and culture of macrophages

Macrophages of bone marrow origin were generated by isolating mononuclear cells flushed from the medullary cavities of femurs and tibias. After collection, cells were gently reconstituted in RPMI-1640 medium containing 25 ng/ml macrophage colony-stimulating factor (R&D Systems, USA) and maintained under standard culture conditions for 7 days to induce differentiation. All these cells were cultured in accordance with established standard protocols [27].

### Primary neuron extraction, culture, and identification

Embryonic mouse neurons were isolated following the manufacturer’s instructions using a commercial neuron extraction kit (Cat. No. 88280, Thermo Fisher Scientific, MA, USA) [28]. Neuronal cells were isolated from mouse embryos at gestational days E16 to E18 for primary culture. Following dissection of spinal cords, dissociation of neurons was achieved through treatment with 0.25% trypsin-EDTA. The enzymatic digestion was halted by the addition of horse serum (Sigma-Aldrich), followed by centrifugation of the resulting cell suspension at 1,000 rpm for 5 min at 4 °C to pellet the cells. The resulting cell pellet was gently resuspended in DMEM (Dulbecco’s Modified Eagle Medium)/F-12 basal medium supplemented with 10% horse serum, 0.5 mM glutamine, and antibiotics (100 IU/ml penicillin and 100 μg/ml streptomycin) to support neuronal viability. Cells were enumerated and seeded onto poly-L-lysine-coated plates (Corning). Once cell attachment was established, the medium was substituted with Neurobasal medium (Gibco) supplemented with1% GlutaMAX, 2% B27, and 1% penicillin–streptomycin to support neuronal growth. Partial medium exchange (50% volume) was carried out every 48 h to support cell viability.

Neurons were identified via immunofluorescence staining, demonstrating expression of NeuN and MAP2 proteins [29].

### In vitro co-culture of cells with hydrogels

In vitro, to assess the effects and cytocompatibility of different hydrogel experimental groups on cells, a co-culture system was employed [30]. Briefly, cells were deposited into the lower chamber of the insert and kept in 1 ml of freshly enriched medium for 12 h. The culture medium was subsequently refreshed with 1 ml of fully supplemented medium, after which 400 μl of hydrogel was introduced into the apical compartment of the Trans-well system to initiate co-culture.

Following the aforementioned co-culture method, neurons were collected for cell viability assessment leveraging a metabolic viability assay (CCK-8), for quantitative evaluation via flow cytometry, and for immunofluorescence-based staining.

Using the aforementioned co-culture method, M1-polarized macrophages stimulated with LPS (lipopolysaccharide) (100 ng/ml) and interferon-γ (IFN-γ; 2.5 ng/ml) were co-cultured separately with PBS, spermidine, the AHAMA hydrogel, and the M-SpdHAMA hydrogel. The collected macrophages were then subjected to flow cytometry and immunofluorescence staining to detect M1 polarization status. Additionally, proteins harvested from cultured cells were analyzed via Western blot.

### In vitro cytotoxicity of the hydrogels

The commercially available Cell Counting Kit-8 (CCK-8) viability assessment (Dojindo, Japan) was utilized to measure intracellular dehydrogenase activity, assessing the impact of AHAMA hydrogels at various concentrations on neuronal activity. Primary neurons were plated at a density of 1.5 × 10^4^ cells per well seeded onto 96-well plates previously conditioned with poly-D-lysine. As a control, PBS solution was used, and following a 24-h incubation period, each well was washed 3 times with PBS solution. Subsequently, a solution containing CCK-8 (10 μl, diluted 1:10) in Neurobasal medium was introduced into every well, with samples kept light-protected at 37 °C for half an hour. Absorbance measurements at 450 nm were obtained for all samples using a Multiskan FC spectrophotometric plate reader (Thermo Scientific, USA). Biological replicates (*n* = 3) were included for all experimental groups. The level of neuronal activity was determined by normalizing dehydrogenase activity in treated samples to that of the PBS group.

### SCI induction in mice and experimental classification

All experimental protocols utilizing animal subjects received authorization from the Institutional Animal Care and Use Committee of Nanjing Medical University and performed in accordance with the National Research Council’s Guide (USA) for the Care and Use of Laboratory Animals. Given that female mice exhibited more anxiety than male mice after SCI, and considering the gender bias in outcomes due to gonadal sex hormones, we selected male mice as the subjects for this study to avoid this bias [31]. Based on preliminary research [32], a murine SCI model was established using male C57BL/6 mice between 8 and 10 weeks of age. Earlier work has validated the use of murine models to explore biomaterial-mediated neural repair following spinal trauma [33]. Following continuous inhalation of isoflurane anesthesia, the dorsal skin of mice was prepared and disinfected. An incision along the midline was followed by staged dissection of the superficial and deep tissues to gain access to the thoracic vertebral column. The dorsal lamina of the T10 vertebra was excised, making the spinal cord readily observable. Upon gaining access to the spinal cord, traumatic modeling was achieved via a calibrated RWD impactor (68097, CA). The cylindrical impactor head, weighing 5 g, had a rounded shape, resulting in a blunt impact on the spinal cord (a standardized lesion was produced via release of a 5-g weight from 6.5 cm using an automated spinal cord contusion apparatus). Muscle and dermal tissues were realigned and sealed with sutures immediately after the impact procedure. Manual bladder emptying was carried out 3 times per day for each subject until normal urinary reflexes were re-established. Experimental animals were divided into separate groups in a randomized manner. Immediately after the establishment of the SCI model, the hydrogels were injected into the injury site using a microsyringe, wherein exposure to 410 nm irradiation at 30 mW cm^−2^ for 60 s facilitated full gel network formation. Layer-by-layer suturing was then performed. All animals were kept under a barrier environment meeting specific-pathogen-free standards.

The specific parameters of the relevant instrument can be found on the official website of the instrument manufacturer (https://www.rwdls.com/).

### Flow cytometry

Neuronal apoptotic profiles were determined via dual fluorescence labeling with Annexin V and propidium iodide, employing a commercially available kit (Cat. No. 556547, BD Biosciences), followed by flow cytometric analysis. After different treatments, the cultured neurons were gently rinsed 2 times with pre-chilled PBS. The harvested pellet was resuspended in assay buffer with 5 μl of Annexin V-FITC (fluorescein isothiocyanate) and 5 μl PI added. The suspension was then incubated for 10 min at room temperature, shielded from light. Cell analysis was conducted through flow cytometry using an FACSVerse 8 instrument (BD Biosciences, Piscataway, NJ, USA), and computational assessment was carried out in FlowJo v7.6.1 platform (Treestar Inc., Ashland, USA).

### Protein immunoblotting technique

Western blot assays followed standardized approaches documented in prior research articles [28]. Cellular proteins were extracted using Beyotime RIPA-based lysis solution (Shanghai, China). Protein quantification was performed via the Bradford assay, followed by SDS-PAGE (sodium dodecyl sulfate–polyacrylamide gel electrophoresis) to resolve equal protein loads, which were electro-transferred onto PVDF (polyvinylidene fluoride) sheets to proceed with immunoblot assays. Following blocking in the presence of 5% BSA (bovine serum albumin) for nonspecific site masking, an overnight binding step with target-specific primary antibodies was performed on the PVDF membranes at 4 °C. The antibodies used included the following: β-actin (1:1,000), cleaved-Caspase-9 (1:500), cleaved-Caspase-3 (1:1,000), Bcl-2 (1:1,000), Bax (1:500), protein tyrosine phosphatase non-receptor 2 (PTPN2; 1:500), STAT1 (1:1,000), phosphorylated STAT1 (1:1,000), and iNOS (1:1,000). After incubation with primary antibodies, horseradish peroxidase (HRP)-linked secondary antibodies (1:10,000) were introduced. Signal development was achieved using enhanced chemiluminescence substrate (ECL; Share-bio, Shanghai, China), and signal intensities from immunoblots were analyzed using ImageJ software provided by NIH (Bethesda, USA).

### Histology and immunofluorescence

Animals underwent intracardiac perfusion using isotonic saline (0.9%) for blood clearance, followed by administration of 4% paraformaldehyde as a fixative. Lesioned spinal cord segments were excised and immersed in 4% paraformaldehyde for overnight fixation. Once tissues were equilibrated in increasing sucrose concentrations (15% then 30%) for cryoprotection, each specimen was encapsulated in a cryoembedding compound (optimal cutting temperature), rapidly frozen, and then sectioned at 10 μm thickness using a cryostat for subsequent histological analysis. Immunofluorescence labeling was carried out on frozen tissue sections. Cryosections of tissue slices were pre-blocked with a 10% solution of BSA to suppress background antigen interactions. To label target proteins, sections were incubated at low temperature (4 °C) overnight with antibodies directed against NF200 (1:100), GFAP (1:200), F4/80 (1:200), and CD86 (1:200). Fluorophore-conjugated secondary antibodies were applied, including a 488 nm-emitting probe targeting mouse IgG (1:500), a 594 nm-conjugated probe specific for rabbit antibodies (1:500), and a Fluor-labeled reagent detectable at 647 nm (1:500). After incubation, excess antibodies were removed by PBS rinsing. DAPI (4′,6-diamidino-2-phenylindole) was applied to visualize nuclei, followed by coverslip placement to prepare samples for fluorescence observation. For consistency, immunofluorescence imaging was captured from equivalent regions within the T10 segment of the injured spinal cords from all mice.

Cells prepared for fluorescence immunostaining underwent fixation in 4% paraformaldehyde (30 min), mild permeabilization with 0.05% Triton X-100, and blocking with 5% BSA to prevent unspecific binding events. For antigen detection, the samples were exposed to iNOS-, NeuN-, MAP2-, and F4/80-directed primary antibodies (each at 1:200 dilution) and maintained at 4 °C for extended binding. The blots were exposed to appropriate HRP-labeled secondary antibodies. Similarly to tissue section immunofluorescence staining, after removal of the secondary antibodies, the cells were washed with PBS and subjected to nuclear staining. Subsequently, an anti-fade mounting medium was applied before fluorescence imaging of the cells.

Fluorescence microscopy data were collected via a Zeiss LSM710 confocal imaging system. ImageJ (NIH, Bethesda, MD, USA) was utilized to evaluate the mean fluorescence intensity across individual fields.

### TUNEL assay

As recommended in the product documentation, detection of apoptotic nuclei was experimentally implemented using a commercial TUNEL (terminal deoxynucleotidyl transferase dUTP nick end labeling) staining kit (T2190-50T, Servicebio, China). Thereafter, nuclear staining was performed with DAPI, and confocal microscopy (LSM710, Zeiss, Germany) was employed to obtain images for evaluation.

### Assessment of locomotor recovery via the Basso Mouse Scale

To determine functional locomotor outcomes after spinal trauma, locomotor ability was graded using the established Basso Mouse Scale (BMS) criteria. Prior to evaluation, all animals underwent habituation training and were individually introduced into an open-field testing environment for behavioral observation [34]. Behavioral scoring was performed by 2 blinded evaluators who were not informed of the experimental grouping. Assessments were conducted at multiple time points post-injury (1, 3, 7, 14, 21, and 28 days). The BMS, ranging from 0 (complete paralysis) to 9 (normal locomotion), evaluates functional recovery by integrating parameters such as hindlimb joint mobility, trunk control, stepping consistency, paw orientation, toe lift, and tail positioning.

### Footprint analysis

Footprint analysis methodology was executed following established protocols, as documented in prior investigations [35]. To distinguish between forepaws and hind paws, blue and red dyes were employed, respectively, for marking the mice’s paws. Subsequently, the footprints generated by the mice as they traversed the paper surface were subjected to observation and subsequent analysis, enabling the assessment of coordination and motor recovery. In brief, mice were first acclimated to the experimental environment for 30 min. Subsequently, mice walked in a straight line on paper, and the dragging of hind paws was assessed. Noticeable drag marks from the hind paws clearly were indicative of suboptimal spinal cord functional restitution. Motor function underwent extended assessment by measuring the step length between the hind paws, with larger step lengths indicating better recovery of spinal cord motor function.

### Electromyography

Motor-evoked potentials were examined in mice using electromyographic analysis 28 days after the injury [36]. Electrical stimulation was applied via electrodes positioned rostrally on the exposed spinal cord segment. Myoelectric responses were recorded from the biceps femoris flexor using surface electrodes, while reference electrodes were secured at distal hindlimb tendinous insertions. A subcutaneous electrode served as the ground to complete the recording circuit.

### The M1 macrophage migration experiment

In the bottom chamber of the Trans-well, an equal volume (500 μl) of PBS, spermidine (100 μM), the AHAMA hydrogel, and the M-SpdHAMA hydrogel was added, followed by supplementation with complete culture medium to a final volume of 1 ml. Subsequently, macrophages were seeded in the upper chamber of the Trans-well, and 500 μm of complete culture medium containing LPS (100 ng/ml) and IFN-γ (2.5 ng/ml) was added. After co-culture for 3 days, the insert was removed, and macrophages on the inner side of the insert were gently wiped off. The outer side cells were then stained with crystal violet, counted, and evaluated for migrated and polarized macrophages (M1).

### Statistical and computational analysis

Statistical computations were performed in Prism 7 (GraphPad Inc., California). All values are reported as mean ± standard deviation (SD), based on a minimum of 3 biologically independent replicates. For datasets involving more than 2 groups, group variations were examined through one-way analysis of variance, and further discrimination between means was performed using Tukey’s test. Unpaired 2-tailed *t* tests were applied to evaluate differences between individual groups. Differences were deemed statistically significant when *P* values fell below the 0.05 threshold.

## Results

### Synthesis and characterization of SpdHAMA

**Fig. 1.**
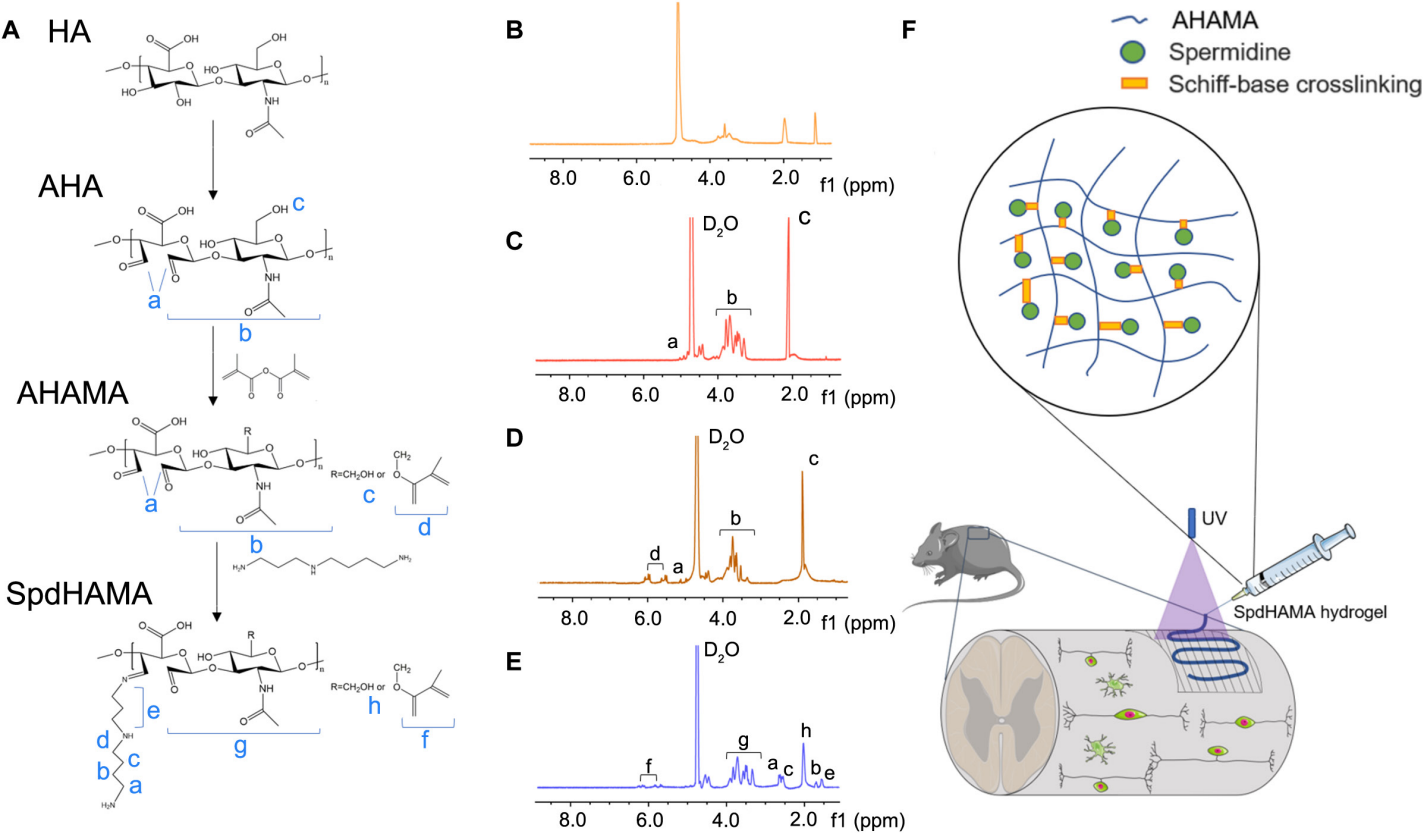
(A) Fabrication process of SpdHAMA hydrogel: Step-by-step synthesis, including aldehyde modification and methacrylation of HA, followed by crosslinking with spermidine via Schiff base reaction and photoinitiator-mediated crosslinking to form SpdHAMA. (B) ^1^H NMR spectrum of HA: Characteristic peaks confirming the intact structure of unmodified HA. (C) ^1^H NMR spectrum of AHA: Peaks confirming successful aldehyde modification of HA. (D) ^1^H NMR spectrum of AHAMA: Peaks indicating successful methacrylation, crucial for light-induced crosslinking. (E) Further characterization of AHAMA: Verification of methacrylate group content and uniform binding to HA. (F) Injection process of SpdHAMA hydrogel: Demonstrates injectability and adaptability for targeted repair in SCI.

To obtain SpdHAMA, the initial step involved the reaction of HA with sodium periodate under precisely controlled conditions, leading to the generation of AHA. Meanwhile, AHA is subjected to a reaction with MA to AHAMA. Subsequently, SpdHAMA was synthesized via a Schiff base coupling process linking spermidine with AHAMA. The fabrication process of SpdHAMA was confirmed by ^1^H NMR (Fig. 1A). In the ^1^H NMR spectra of both AHA and AHAMA, characteristic resonance signals were identified at approximately 5.0 and 5.2 parts per million (ppm), corresponding to hemiacetal protons generated by the interaction between aldehyde functionalities and adjacent hydroxyl groups (Fig. 1C and D). Analysis of the ^1^H NMR data for AHAMA revealed characteristic signals at approximately 5.6 and 6.0 ppm, attributable to protons of the MA C=C group (Fig. 1D). Successful incorporation of spermidine into the AHAMA backbone was verified by ^1^H NMR spectroscopy, evidenced by distinct methylene resonance signals at δ 1.4, 1.5, 2.5, and 2.6 ppm, corresponding to the –CH₂– groups of spermidine (Fig. 1E). The FTIR spectra demonstrated that unmodified HA exhibited a broad and intense absorption peak within the range of 3,200 to 3,600 cm^−1^, indicating an abundance of hydroxyl (–OH) groups. The spectra also revealed asymmetric and symmetric stretching vibrations of carboxylate groups at 1,600 and 1,400 cm^−1^, respectively, along with vibration peaks of C–O–C and C–OH bonds within the sugar ring, detected in the range of 1,000 to 1,200 cm^−1^, representing typical characteristic peaks of HA (Fig. S1A). For AHA, a new absorption peak emerged near 1,730 cm^−1^, indicative of carbonyl (C=O) stretching vibrations, confirming that successful introduction of aldehyde groups. Concurrently, the broad hydroxyl peak weakened slightly, indicating partial oxidation of hydroxyl groups (Fig. S1B). In the spectrum of AHAMA, the presence of a C=C double bond stretching vibration at 1,630 cm^−1^ and an ester C=O absorption peak at 1,720 cm^−1^ verified the successful methacrylation. The hydroxyl peak further weakened, suggesting more extensive chemical modification (Fig. S1C). For spermidine-modified hyaluronic acid (SpdHAMA), a clear stretching vibration signal of the C=N bond was observed at 1,620 cm^−1^, indicating the formation of Schiff base bonds between spermidine and the aldehyde groups. Additionally, N–H stretching vibrations were detected in the range of 3,200 to 3,500 cm^−1^. The C=O peak at 1,730 cm^−1^, which was prominent in AHA, was significantly reduced in SpdHAMA, further confirming the involvement of aldehyde groups in the grafting reaction with spermidine (Fig. S1D).

To further validate the chemical modifications and assess the resulting changes in polymer structure, GPC was conducted following FTIR analysis. The molecular weight distributions of HA, AHA, AHAMA, and SpdHAMA were conducted using GPC mode. The chromatograms and molecular weight parameters, including the number-average molecular weight (*M*_n_), weight-average molecular weight (*M*_w_), and dispersity index (*Đ*), are summarized in Table 1 and presented in Fig. S2. After periodate oxidation to obtain AHA, *M*_n_ and *M*_w_ increased to 80,145 and 116,920 g/mol, respectively, with a slightly higher dispersity (*Đ* ≈ 1.46). Further methacrylation of AHA to produce AHAMA resulted in an *M*_n_ of 126,933 g/mol and an *M*_w_ of 246,942 g/mol, with the dispersity increasing to 1.95, indicating a broader molecular weight distribution due to the introduction of MA groups. Spermidine was grafted onto AHAMA via a Schiff base reaction to yield SpdHAMA, with *M*_n_ and *M*_w_ increasing significantly to 148,626 and 565,076 g/mol, respectively, and the dispersity reaching 3.80, suggesting a more complex structure and broader molecular weight distribution after multiple modification steps. The fitted molecular weight distribution curves in Fig. S2 clearly showed a shift toward higher molecular weights from HA to SpdHAMA, along with a broadening of the distribution profile, consistent with the increasing trend of the dispersity index (*Đ*). An increase in dispersity reflected expanded molecular weight variability and the coexistence of diverse polymeric species in the sample. This phenomenon may be attributed to variations in the degree of methacrylation and the efficiency of subsequent Schiff base modification, as well as potential differences in the reaction efficiency of the HA backbone during each modification step.

**Table 1.** Molecular weight parameters of HA, AHA, AHAMA, and SpdHAMA measured by GPC. *M*_n_ represents the number-average molecular weight, *M*_w_ is the weight-average molecular weight, and *Đ* is the dispersity index (*M*_w_/*M*_n_).

Sample	*M*_n_ (g/mol)	*M*_w_ (g/mol)	*Đ*
HA	66,980	92,630	1.38
AHA	80,145	116,920	1.46
AHAMA	126,933	246,942	1.95
SpdHAMA	148,626	565,076	3.80

**Fig. 2.**
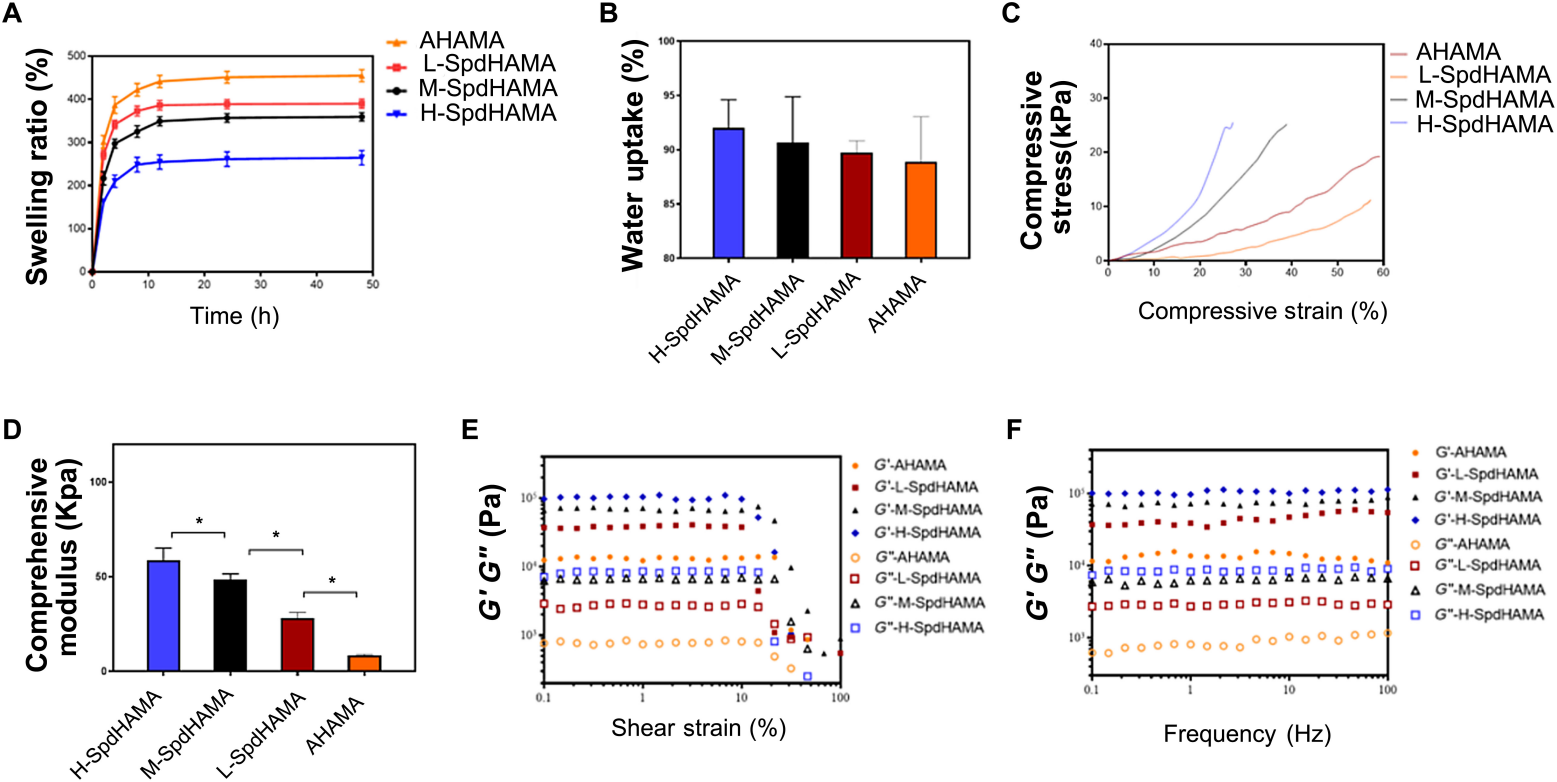
(A) The swelling ratio of AHAMA. (B) The water uptake of the SpdHAMA hydrogels. (C) The stress–strain curve of SpdHAMA hydrogels. (D) The Young’s modulus of SpdHAMA hydrogels. (E) The linear viscoelastic (LVE) range of the gel. (F) Gelation kinetics assessed by time-dependent rheology under 1% strain.

To create a SpdHAMA hydrogel, a solution containing 5.0% (w/v) SpdHAMA and 0.3% (w/v) photoinitiator LAP was prepared in PBS. Subsequently, the solution was subjected to 30 mW cm^−2^ UV light (410 nm) for 1 min, achieving the photocrosslinking step. The SpdHAMA hydrogel adheres effectively to the injured tissue, facilitating improved wound coverage and integration (Fig. 1F). The samples were designated as L-SpdHAMA, M-SpdHAMA, and H-SpdHAMA, corresponding to the respective spermidine concentrations of 1, 2, and 4 mM. The swelling and water uptake properties of different hydrogels were evaluated by swelling ratio and water uptake test. Swelling ratio of all hydrogels showed rapid increase and reached a plateau within 8 h. The swelling ratio of AHAMA, L-SpdHAMA, M-SpdHAMA, and H-SpdHAMA was 264.53% ± 16.82%, 358.18% ± 9.96%, 389.85% ± 10.61%, and 454.89% ± 14.03%, respectively (Fig. 2A). The water uptake of the SpdHAMA hydrogels is shown in (Fig. 2B). The water uptake of AHAMA, L-SpdHAMA, M-SpdHAMA, and H-SpdHAMA was 88.89% ± 4.15%, 89.76% ± 1.055%, 90.67% ± 4.208%, and 92.02% ± 2.580%, respectively. Comparative analysis revealed no statistically meaningful variation across the 4 groups. The variation in swelling ratio can be attributed to the elevated crosslinking density, which is in agreement with the observed reduction in pore size within the hydrogel matrix. This effect arises from dynamic Schiff-type bonds between aldehyde moieties on AHAMA chains and amine functionalities present in spermidine, resulting in a denser network of physical crosslinking sites.

Compressive strength was measured across hydrogel samples using standardized compression protocols. The stress–strain curve was performed to explore the relation between compressive strain and stress (Fig. 2C). Mechanical test showed that the Young’s modulus was significantly higher in the H-SpdHAMA hydrogel than that in M-SpdHAMA. The Young’s modulus of M-SpdHAMA was higher than that of L-SpdHAMA. The compressive modulus of AHAMA, L-SpdHAMA, M-SpdHAMA, and H-SpdHAMA were 8.59% ± 0.50%, 28.26% ± 2.90%, 48.44% ± 3.01%, and 58.69± 6.42%, respectively (Fig. 2D). To characterize the rheological behavior of the optimized hydrogel, a series of dynamic oscillatory tests were conducted. Initially, oscillatory strain measurements were carried out to define the LVE region, which defines the deformation range wherein the material maintains structural integrity (Fig. 2E). The LVE range was determined to span from 0.1% to 10% strain, beyond which structural failure was observed. Subsequently, a time sweep analysis at 1% strain was carried out to assess the gelation kinetics under stable deformation conditions. The dynamic frequency sweep technique is employed for assessing the rheological properties of various hydrogels (Fig. 2F). Throughout the tested frequency range, the storage modulus (*G*′) consistently exceeded the loss modulus (*G*″), demonstrating the predominance of elastic behavior and confirming the successful assembly of a chemically bonded gel framework. The *G*′ values and *G*″ values increased dependent on spermidine concentration, suggesting that Schiff base bond could enhance the mechanical modulus of the hydrogel.

**Fig. 3.**
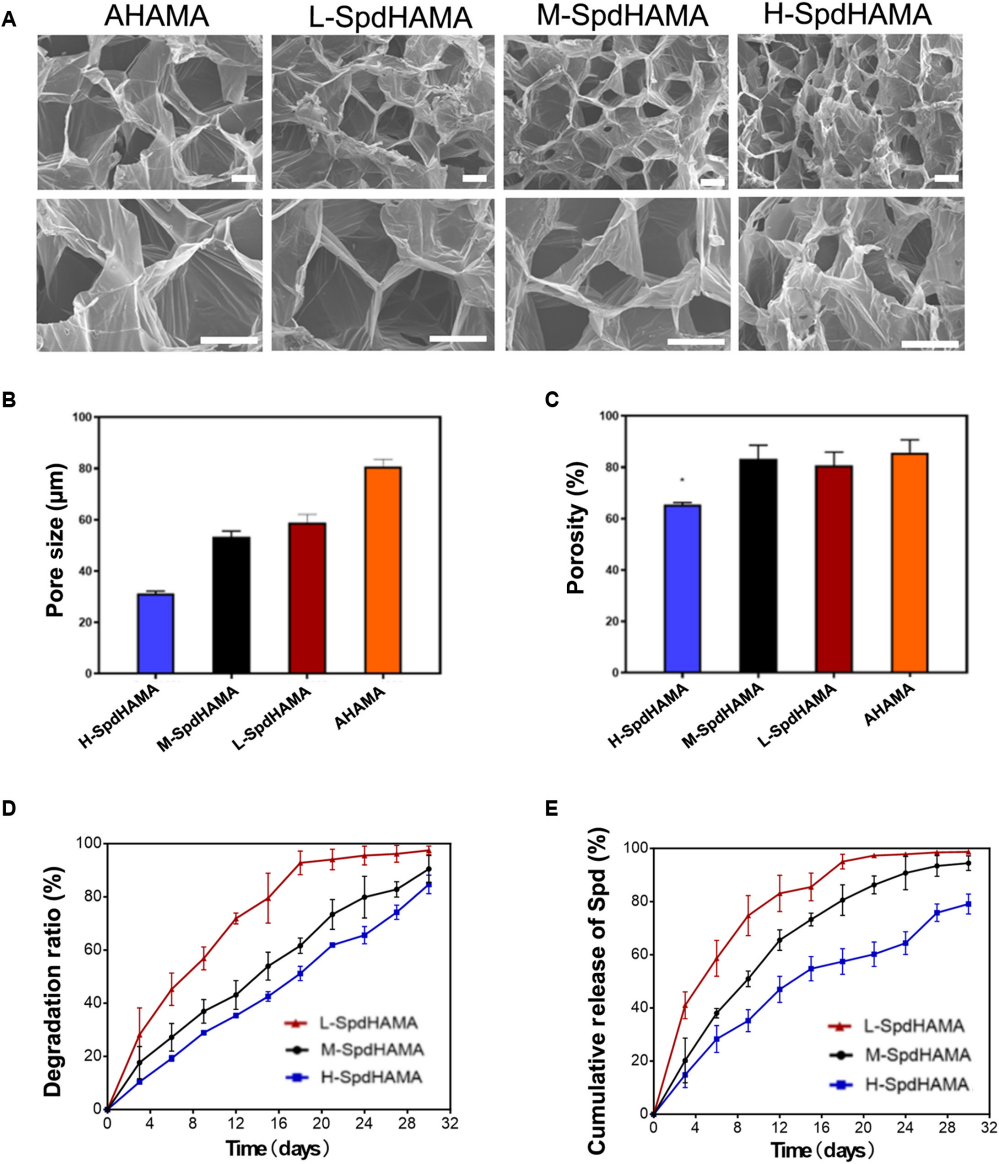
(A) SEM-based imaging of distinct SpdHAMA hydrogel formulations. (B) The pore sizes of different hydrogels. (C) The porosities of different hydrogels. (D) In vitro determination of hydrogel degradation profiles across the observation period (*n* = 5). (E) Cumulative release trajectory of spermidine in vitro (*n* = 5).

The microarchitectural features of SpdHAMA hydrogels were visualized using SEM (Fig. 3A). With the spermidine content increased, the SEM shows a denser structure and a smaller pore size. The pore sizes of AHAMA, L-SpdHAMA, M-SpdHAMA, and H-SpdHAMA were 80.88 ± 2.737 μm, 59.01 ± 3.10 μm, 53.35 ± 2.01 μm, and 31.32 ± 0.86 μm (Fig. 3B). The porosities of AHAMA, L-SpdHAMA, M-SpdHAMA, and H-SpdHAMA were 85.76% ± 4.94%, 80.74% ± 5.17%, 83.38% ± 5.23%, and 65.46% ± 0.83% (Fig. 3C). With increasing spermidine concentration, the pore size decreases. This phenomenon arises from Schiff base coupling between the aldehyde groups on AHAMA molecular chains and the amino groups on spermidine, leading to the formation of additional, denser physical crosslinking points. These findings demonstrated the successful coupling of spermidine to AHAMA. The addition of spermidine at varying concentrations has an impact on the mechanical properties, pore size, and porosity of the hydrogel. Pore diameters ranging from 50 to 100 μm offered sufficient 3-dimensional architecture and surface interface to support cellular infiltration and attachment. To maximize spermidine loading capacity while maintaining hydrogel porosity and pore size, as well as preserving cell adhesion and migration, M-SpdHAMA was identified as the optimal formulation compared to other hydrogel types.

To evaluate the in vitro degradation properties of SpdHAMA hydrogels with different spermidine concentrations, we analyzed the degradation rates of L-SpdHAMA, M-SpdHAMA, and H-SpdHAMA over 28 days (Fig. 3D). The results demonstrated a progressively increasing degradation trend across all 3 hydrogel formulations. L-SpdHAMA exhibited the fastest degradation rate, with approximately 98% degradation by day 28, whereas M-SpdHAMA degraded by approximately 80%. In contrast, H-SpdHAMA showed the slowest degradation, with a degradation rate of ~65% on day 28. These findings indicate that the degradation kinetics of SpdHAMA hydrogels are significantly influenced by spermidine concentration, with higher spermidine levels leading to slower degradation, likely due to increased crosslinking density, which enhances hydrogel structural stability.

We further investigated the in vitro spermidine release profiles of different SpdHAMA hydrogel formulations (Fig. 3E). The cumulative release rate of L-SpdHAMA reached approximately 98% by day 28, exhibiting a rapid release profile, whereas M-SpdHAMA and H-SpdHAMA displayed cumulative release rates of ~79% and ~65%, respectively. The release rate of spermidine was closely correlated with the degradation kinetics of these hydrogels: L-SpdHAMA exhibited a higher release rate due to its faster degradation, whereas H-SpdHAMA demonstrated a lower release rate due to its slower degradation profile. Overall, the M-SpdHAMA hydrogel achieved a relatively balanced degradation-release profile, making it a promising candidate for sustained drug delivery applications.

To further investigate the release mechanisms of SpdHAMA hydrogels, we performed kinetic modeling using the first-order kinetic model, Higuchi model, and Korsmeyer–Peppas model to fit the release profiles of L-SpdHAMA, M-SpdHAMA, and H-SpdHAMA (Table 2). The fitting results demonstrated that the first-order kinetic model provided the best fit for M-SpdHAMA and L-SpdHAMA, with *R*^2^ values of 0.9985 and 0.9955, respectively, indicating that their release profiles were primarily degradation-driven, conforming to first-order kinetics. In contrast, the Korsmeyer–Peppas model best described the release behavior of H-SpdHAMA, with an *R*^2^ value of 0.9894, suggesting that multiple mechanisms may be involved in its release process. These findings indicate that modulating spermidine concentration enables precise tuning of the release characteristics of SpdHAMA hydrogels, with different hydrogel formulations exhibiting distinct release mechanisms.

**Table 2.** Release kinetics fitting analysis

Sample	Model	Parameters	*R*^2^ value
M-SpdHAMA	First-order kinetic	C_0 _= 107.92, *k* = 0.0746	0.9985
	Higuchi	k_H = 18.0392	0.9761
	Korsmeyer–Peppas	K_p_ = 15.0862, *n* = 0.5604	0.9807
H-SpdHAMA	First-order kinetic	C_0_ = 95.77, *k* = 0.0530	0.9885
	Higuchi	k_H = 13.5621	0.9689
	Korsmeyer–Peppas	K_p_ = 8.9186, *n* = 0.6411	0.9894
L-SpdHAMA	First-order kinetic	C_0_ = 99.72, *k* = 0.1537	0.9955
	Higuchi	k_H = 20.8409	0.9209
	Korsmeyer–Peppas	K_p_ = 33.7486, *n* = 0.3359	0.9765

The results indicate that the M-SpdHAMA hydrogel enables sustained and stable release of spermidine. Based on comprehensive analyses, including degradation kinetics, release profiles, and release kinetics modeling, M-SpdHAMA emerges as the preferred formulation for controlled-release therapy in SCI treatment.

### AHAMA hydrogels demonstrate no neuronal toxicity

The cytocompatibility of AHAMA hydrogels was assessed by quantifying dehydrogenase activity in primary neurons (Fig. S3). Over the investigated dosing interval (0.1 to 10.0 mg/ml) and incubation timeline (6 to 96 h), no detectable cytotoxic effects were observed, indicating favorable neuronal compatibility of the material.

Interestingly, prolonged incubation with AHAMA hydrogels led to a time-dependent increase in neuronal activity. At 24 h, a pronounced viability increase appeared exclusively at 10.0 mg/ml, the top concentration examined. However, after 48 h of exposure, even low concentrations (as low as 1.0 mg/ml) elicited a marked increase in neuronal viability (Fig. S3A). For the M-SpdHAMA hydrogel, no neurotoxicity was detected across the concentration range of 1.0 to 10.0 mg/ml. Furthermore, neuronal activity was significantly enhanced following co-culture with the M-SpdHAMA hydrogel across doses between 0.1 and 10.0 mg/ml combined with exposure durations of 6 to 96 h (Fig. S3B).

**Fig. 4.**
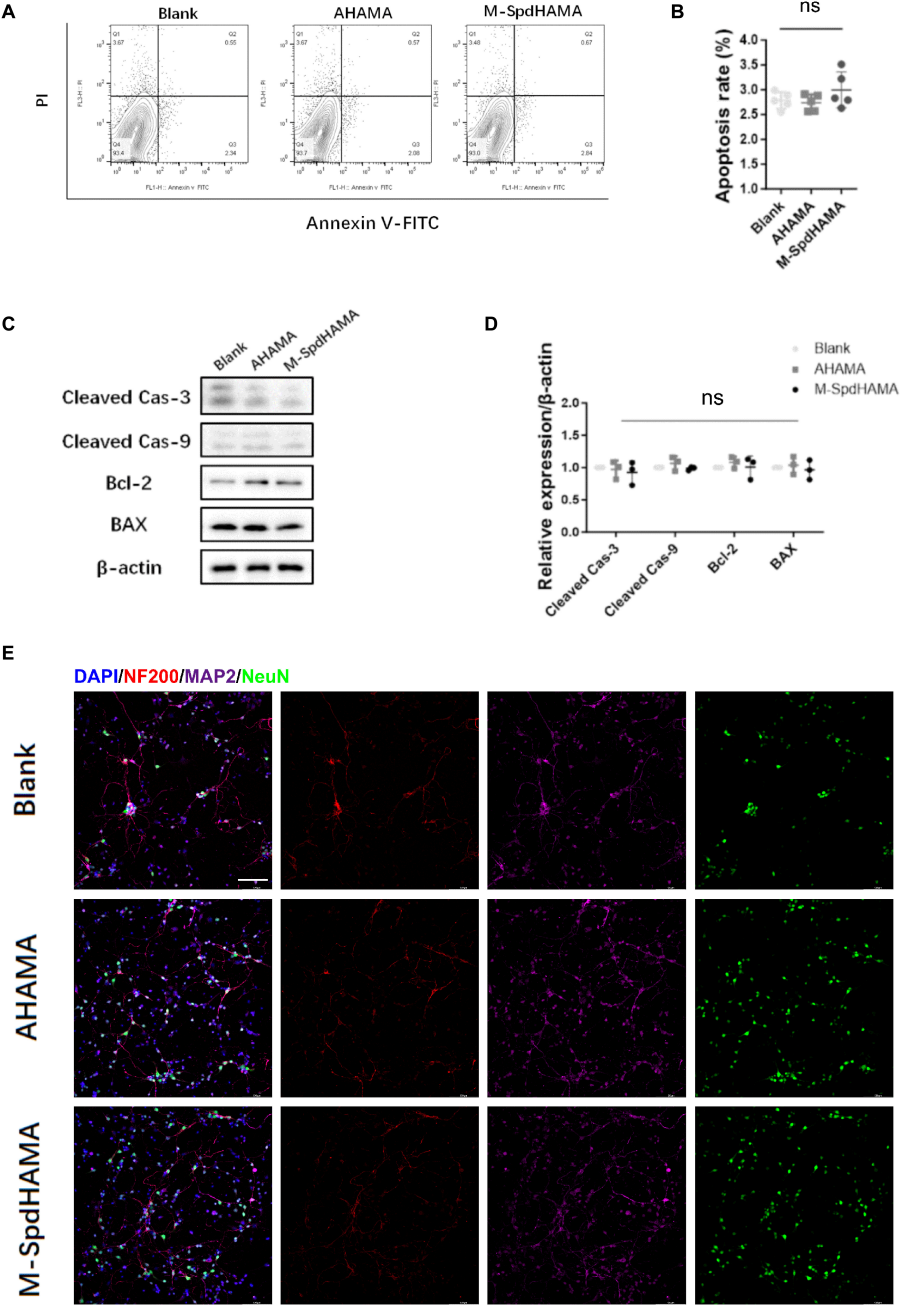
Neuronal compatibility of AHAMA hydrogels. (A and B) Flow cytometry analysis and quantitative results of neurons undergoing apoptosis by PI and Annexin V-FITC labeling (*n* = 5). (C) Representative Western blot-based evaluation of apoptosis-related proteins in neurons treated with AHAMA or M-SpdHAMA hydrogels. (D) Quantitative immunoblot analysis of neuronal apoptotic markers with β-actin as a loading control (*n* = 3). (E) Representative immunofluorescence staining images of NeuN (in green) and MAP2 (in red) in primary neurons (*n* = 5; scale bar: 100 μm). ns, not statistically significant.

In order to further validate the non-toxicity of AHAMA hydrogels toward neurons, we conducted ex vivo co-cultivation of primary mouse neurons with AHAMA hydrogels. Flow cytometry analysis revealed no significant differences in apoptosis between the co-cultured AHAMA hydrogel group (AHAMA group), the M-SpdHAMA hydrogel group (M-SpdHAMA group), and the control group (Blank group) (Fig. 4A and B). Immunoblot detection of apoptosis-associated factors demonstrated negligible differences across treatments among the AHAMA group, M-SpdHAMA group, and Blank group (Fig. 4C and D). Live/dead staining of co-cultured neurons revealed no significant differences in neuronal survival among the Blank group, AHAMA group, and M-SpdHAMA group (Fig. S4A and B). Furthermore, immunofluorescence staining showed no noticeable morphological differences among neurons in the 3 groups. Neuronal abundance was notably higher in the M-SpdHAMA group versus Blank, while AHAMA and Blank groups remained statistically indistinguishable (Fig. 4E and Fig. S4C). These findings collectively suggest that AHAMA hydrogels exerts no toxic effects on neurons and demonstrates excellent biocompatibility.

### SpdHAMA hydrogels provide protection for SCI

**Fig. 5.**
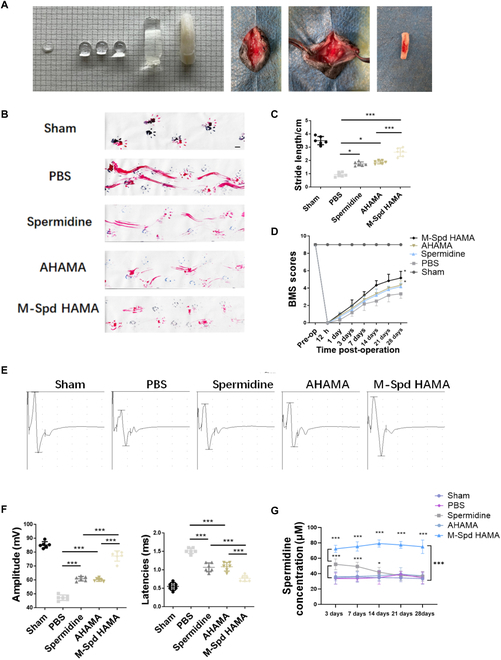
SpdHAMA hydrogels safeguard the spinal cord following traumatic injury. (A) The physical properties of SpdHAMA hydrogels after solidification and the characteristics of hydrogels coagulated and adhered at SCI sites. (B and C) Footprint analysis of each group (Sham, Sham operation group; PBS, the phosphate-buffered saline group; AHAMA, the AHAMA hydrogel group; M-SpdHAMA, the moderate spermidine-conjugated AHAMA group) and quantification performed 28 days subsequent to SCI induction. Blue coloration corresponds to the anterior paw prints; red represents posterior paw prints (*n* = 6). (D) Motor function was tracked via BMS evaluations across the 28-day recovery period following SCI under different interventions (*n* = 6). (E and F) Electromyographic analysis of hind limb motor function and quantitative assessment in mice from each treatment group at 28 days post-SCI (*n* = 6). (G) Concentration of spermidine in mouse cerebrospinal fluid (*n* = 3). *P < 0.05; ***P < 0.001.

In light of spermidine’s recognized protective influence on neural tissue and AHAMA’s supportive interaction with neurons, we have developed SpdHAMA hydrogels for treating post-traumatic spinal cord injuries. Following UV irradiation, SpdHAMA hydrogels demonstrated favorable physical attributes, thereby facilitating robust adherence with the site of SCI (Fig. 5A). On post-injury day 28, motor behavior was monitored via footprint pattern analysis to evaluate locomotor restoration (Fig. 5B). Based on statistical analysis of footprint drag and stride length, both the intrathecal injection of spermidine and exclusive AHAMA hydrogels treatment in SCI exhibited significant functional recovery. However, neither of these groups demonstrated a therapeutic capacity for SCI repair as pronounced as the SpdHAMA hydrogel group (Fig. 5B and C). Further evidenced by the BMS scores, throughout the process of SCI recovery, the SpdHAMA hydrogel group exhibited a notably superior therapeutic effect for SCI treatment, with significant limb function recovery compared to both the intrathecal injection of spermidine group and the AHAMA hydrogel group, especially at post-injury day 28 (Fig. 5D). Electromyographic analysis of mouse hind limb muscles also revealed the superior therapeutic efficacy of the SpdHAMA hydrogel group in facilitating enhanced recovery of limb motor function subsequent to SCI (Fig. 5E and F). The aforementioned findings collectively underscore that the isolated utilization of either spermidine or AHAMA hydrogels exhibits a certain level of therapeutic efficacy against SCI. Nevertheless, this impact remains circumscribed. By contrast, the preparation of the SpdHAMA hydrogel offers a more potent avenue for the treatment of SCI, notably fostering a more robust reinstatement of spinal motor function.

We collected CSF from mice and measured spermidine concentrations at different time points in various treatment groups to investigate the sustained drug liberation properties of SpdHAMA hydrogel. Our findings revealed that the group receiving intrathecal injection of spermidine exhibited initially higher concentrations in CSF but failed to maintain them. In contrast, the SpdHAMA hydrogel group maintained elevated spermidine concentrations throughout the entire treatment process (Fig. 5G), indicating the hydrogel’s capacity for effective spermidine sustained release and maintenance of a certain high concentration within the spinal cord microenvironment.

### SpdHAMA hydrogels promote neuronal recovery in SCI by inhibiting M1 macrophage polarization

**Fig. 6.**
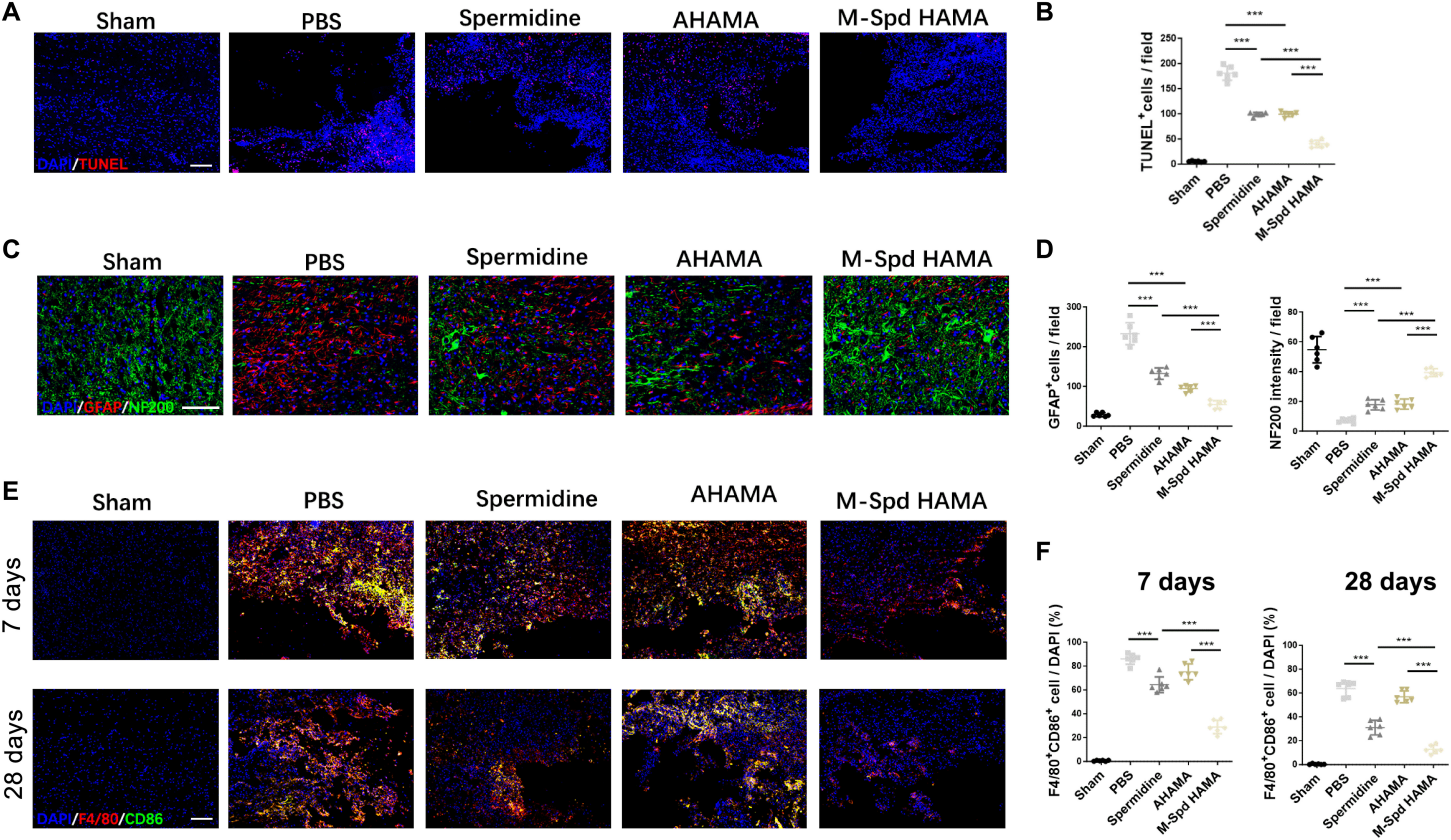
SpdHAMA hydrogels enhance neuronal recovery in SCI by restraining M1 macrophage polarization. (A and B) TUNEL staining and quantitative analysis revealed apoptotic cell (TUNEL^+^ cell) numbers in various treatment groups (*n* = 6; scale bar: 200 μm). (C and D) Immunofluorescence staining analysis elucidated the distribution of neurons (NF200, green) and astrocytes (GFAP, red) in different treatment groups at 28 days post-SCI (*n* = 6; scale bar: 100 μm). (E and F) Comparative immunofluorescence evaluation of M1 macrophage polarization in distinct therapeutic cohorts during early (day 7) and late (day 28) stages of SCI recovery (*n* = 6; scale bar: 200 μm). ***P < 0.001.

Limiting apoptosis and enhancing neuronal repair constitute essential determinants of motor recovery after spinal cord trauma [37]. Exploring the neuroprotective effects of SpdHAMA hydrogels on SCI, tissue sections obtained 28 days post-SCI, subjected to TUNEL staining, revealed a significant reduction in apoptotic cells in the treatment groups employing spermidine and the hydrogel alone, in comparison to the untreated PBS group (Fig. 6A and B). Further investigation indicated that, when treated with SpdHAMA hydrogels, a significant decrease in apoptotic cells was observed in comparison to both the spermidine-only group and the AHAMA hydrogel-only group (Fig. 6A and B). This suggests that in SCI, the sustained-release spermidine hydrogel exhibits enhanced neuroprotective properties, leading to reduced cellular apoptosis. Immunofluorescence staining elucidated the distribution of neurons (NF200) and astrocytes (GFAP) in the spinal cord. In the absence of treatment (PBS group), astroglial populations showed significant enlargement, whereas neuronal distribution was concurrently reduced. In cases of poor SCI recovery, diminished neuronal distribution was evident, accompanied by an increase in proliferating astrocytes (Fig. 6C and D). The solitary application of spermidine or the AHAMA hydrogel partially ameliorated this injury, fostering neuronal recovery to a certain extent and attenuating astrocytic proliferation, yet with limitations. Immunofluorescence findings suggested that the SpdHAMA hydrogel group, compared to other treatment cohorts, exhibited a superior capacity to restore neural network architecture and curtail astrocytic proliferation (Fig. 6C and D).

Interestingly, employing immunofluorescence staining, we observed a remarkable reduction in M1 macrophage polarization in spinal cord injuries treated with the SpdHAMA hydrogel compared to the other treatment groups. In mice SCI, macrophage migration and aggregation predominantly peak at 7 days post-injury [38]. By the 7th day post-injury, compared to other treatment groups, the SpdHAMA hydrogel significantly reduces polarization and aggregation of M1 macrophages. Additionally, the inhibitory effect of this material persists until the late stage of injury repair, extending to day 28 post-injury (Fig. 6E and F). This finding strongly suggests that the SpdHAMA hydrogel’s capacity to significantly protect neurons is likely attributed, in part, to this substantial decrease in M1 macrophage polarization.

### SpdHAMA hydrogels inhibit M1 macrophage polarization through the PTPN2–STAT1 axis

**Fig. 7.**
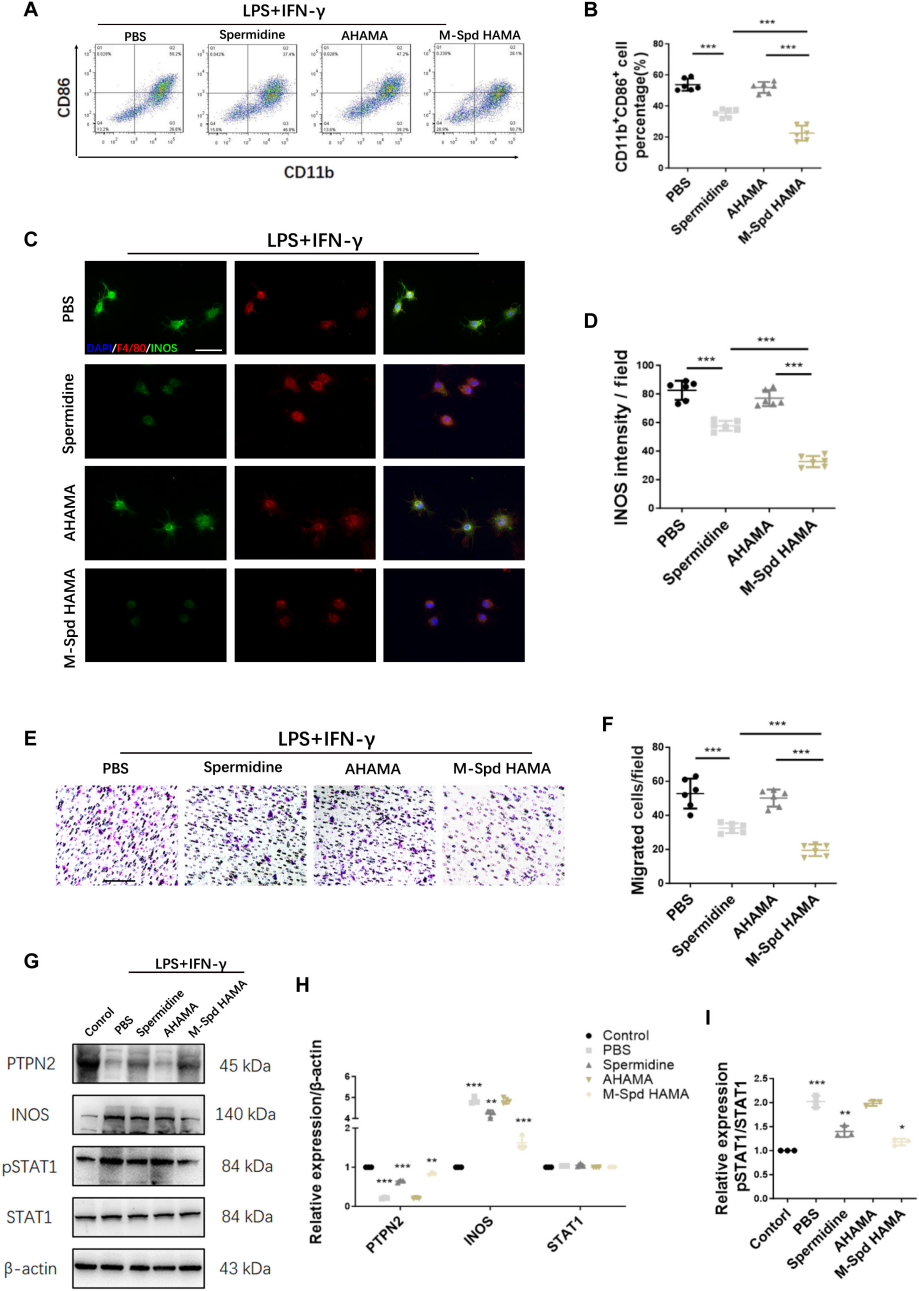
SpdHAMA hydrogels affect M1 macrophage polarization via modulation of the PTPN2–STAT1 pathway. (A and B) Flow cytometry dot plots and relative percentage of CD11b^+^ and CD86^+^ cells (the M1 macrophage subset) across different treatment groups (*n* = 6). (C) Representative immunofluorescence images of M1 macrophages (INOS, green; F4/80, red) in different treatment groups (*n* = 6; scale bar: 20 μm). (D) Quantification of mean fluorescence intensity of INOS in different treatment groups (*n* = 6). (E and F) Trans-well assay images and quantitative analysis of the migration of M1 macrophages in different treatment groups (*n* = 6; scale bar: 100 μm). (G to I) Western blot analysis quantifying the relative expression levels of PTPN2, iNOS, total STAT1, and phosphorylated STAT1 in macrophages across various therapeutic regimens (*n* = 3). *P < 0.05; **P < 0.01; ***P < 0.001.

Spermidine serves as an essential regulator of sustained inflammation underlying neurodegenerative disorders [16]. Additionally, we observed significant neuroprotective effects and inhibition of M1 macrophage polarization upon SpdHAMA hydrogels for SCI treatment. To dissect the regulatory mechanisms governing M1 polarization inhibition induced by SpdHAMA hydrogels, we performed co-cultures using primary mouse bone marrow macrophages extracted in vitro. In vitro, macrophages polarized into M1 phenotype through stimulation with LPS and IFN-γ [39]. Flow cytometry experiments revealed that spermidine could mitigate M1 macrophage polarization, whereas AHAMA hydrogels alone did not exhibit polarization inhibition. However, a more pronounced effect in suppressing M1 macrophage polarization was observed when macrophages were treated with SpdHAMA hydrogels. This might be attributed to the gradual release of spermidine facilitated by AHAMA hydrogel degradation (Fig. 7A and B). Immunofluorescence cell staining further substantiated the significant role of SpdHAMA hydrogels in inhibiting M1 macrophage polarization. In comparison to other co-culture groups, the SpdHAMA hydrogel group exhibited reduced expression of INOS in M1 macrophages and displayed cellular morphologies that leaned toward non-M1 polarization states (Fig. 7C and D). Furthermore, Trans-well migration assays demonstrated that spermidine significantly reduced the migration of M1 macrophages versus its PBS counterpart. The SpdHAMA hydrogel group exhibited even more efficient suppression of M1 macrophage migration compared to the spermidine-only group (Fig. 7E and F).

PTPN2 stands as a pivotal protein governing immune cells, including T cells, B cells, and macrophages [40]. Serving as a signaling molecule, PTPN2 exhibits the capacity to modulate a multitude of signaling pathways, encompassing prominent targets such as EGFR (epidermal growth factor receptor), CSF1R (colony-stimulating factor 1 receptor), and IR (insulin receptor), as well as non-receptor protein tyrosine kinases, such as Src (Src family kinases) and STAT family kinases. Consequently, in pursuit of unraveling the molecular mechanisms underlying spermidine-mediated regulation of M1 macrophage polarization, we conducted Western blot analysis on macrophages from diverse treatment cohorts to validate pertinent proteins. We observed that under non-polarized treatment conditions in macrophages, PTPN2 exhibited significantly higher expression levels relative to the PBS-treated control. This finding highlights PTPN2 as a pivotal modulator of M1 macrophage polarization. Notably, elevated expression of PTPN2 correlated with suppressed phosphorylation of STAT1 and a reduction in INOS markers, ultimately leading to the inhibition of M1 macrophage polarization (Fig. 7G to I). When polarized M1 macrophages were exclusively subjected to AHAMA treatment, no detectable shift in PTPN2 expression was evident, and polarization-related markers remained unaltered. However, when treated solely with spermidine or subjected to SpdHAMA hydrogel treatment, a marked increase in PTPN2 expression was evident as opposed to the PBS control condition. Furthermore, the SpdHAMA hydrogel elicited a stronger induction of PTPN2 than free spermidine, while concurrently attenuating STAT1 phosphorylation and reducing iNOS abundance (Fig. 7G to I). The above results elucidate the molecular mechanisms by which spermidine influences the targeted regulation of M1 macrophage polarization through the PTPN2-mediated modulation of the STAT1 signaling axis. Additionally, these findings underscore that spermidine gel formulations effectively releases spermidine, ensuring a sustained and stable inhibition of M1 macrophage polarization.

## Discussion

SCI inflicts extensive functional impairments, profoundly diminishing the quality of life of those affected. Presently, clinical interventions still lack effective therapeutic strategies for SCI [1]. At present, clinical interventions continue to lack efficacious therapeutic modalities for the treatment of SCI. Conventional clinical approaches encompass hormone-based pulse therapy, spinal cord decompression surgery, and rehabilitation regimens, yet their efficacy in restoring motor and sensory functions in recovering patients remains exceedingly limited [41]. Injectable hydrogels have emerged as powerful candidates for driving progress in tissue repair and engineering, supported by a wealth of experimental evidence [42]. Therefore, in this study, for the first time, we prepared AHAMA hydrogels and employed a Schiff base reaction with spermidine to synthesize a photocrosslinked injectable SpdHAMA hydrogel. The composite exhibited excellent mechanical properties and biological activity. The SpdHAMA hydrogel is essentially composed of HA, a natural component of the ECM. This enables the hydrogel to effectively mimic the native ECM conditions, thereby providing a dynamic environment conducive to cell proliferation and growth. Furthermore, the sustained release of spermidine inhibits the M1 polarization of macrophages during SCI, thereby promoting the repair of SCI. Therefore, the injectable SpdHAMA hydrogel developed in this study may facilitate the enhancement of functional recovery in SCI and offer crucial experimental groundwork for the clinical application of injectable hydrogels in SCI treatment.

By crosslinking methacrylated HA with variable spermidine loadings, a hydrogel system capable of sustained spermidine delivery was prepared. The AHAMA hydrogel can enhance the drawbacks associated with HA hydrogels as biomaterials, including poor mechanical properties, weak cell adhesion, and high degradation rates [43]. This AHAMA hydrogel forms a porous structure and absorbs a significant amount of water, thereby providing oxygen and nutrients for cell growth. Due to the MA substitution, the hydrogel possesses strong mechanical strength, making it suitable for use as a tissue filler in biological tissue repair engineering. Furthermore, through the dynamic Schiff base reaction coupled with spermidine and simultaneous reaction with the photo-initiator LAP, light-crosslinked SpdHAMA hydrogels were prepared. In the preparation of biomaterials, a critical factor is controlling reaction conditions to ensure both the hydrogel’s physical strength and interaction with biological systems. Earlier investigations indicate that variations in reaction temperature and duration critically modulate crosslinking efficiency, ultimately shaping the hydrogel’s physicochemical characteristics [44]. In this study, we fine-tuned the crosslinking density by adjusting the spermidine concentration. Increasing spermidine content reinforced crosslink formation, substantially augmenting the hydrogel’s mechanical stability. The selection of spermidine concentration was based on its physiological effects reported in prior research, as well as the release profiles of spermidine observed in in vitro experiments. Moreover, moderate concentrations of spermidine promote tissue regeneration by regulating cell growth and mitigating oxidative stress. Therefore, we chose a range of spermidine concentrations to evaluate their impact on neural regeneration and hydrogel degradation, providing data to optimize dosage for future clinical applications.

Physicochemical features of hydrogels dictate their functional capacity in regenerative applications. According to our testing results, higher spermidine concentrations led to hydrogels exhibiting greater mechanical strength and slower degradation rates, which is consistent with the positive correlation between crosslinking density and mechanical properties. Enhanced mechanical properties are critical for early spinal cord repair, as they provide necessary physical support and help prevent secondary injury. Furthermore, the pore structure of the hydrogel is also influenced by spermidine concentration. Smaller pore sizes help limit the infiltration of inflammatory cells, while expanded pore architecture favors cell motility and stimulates vascularization [45]. Therefore, we hypothesize that different stages of spinal cord repair may require hydrogels with different pore sizes. Through experiments including swelling ratio test, water uptake test, compressive testing, and rheological analysis, conducted to assess SpdHAMA hydrogels at varying concentrations of spermidine, we observed that the M-SpdHAMA hydrogel exhibited optimal characteristics. Our research indicates that the incorporation of spermidine significantly enhances the mechanical resilience of the engineered hydrogel. Increasing the content of spermidine leads to improvements in the mechanical performance of the hydrogel, but it also alters the pore size and porosity of the hydrogel. Additionally, SEM hydrogel sample preparation was conducted using lyophilization, and this process can impact pore sizes due to the freezing process. However, measuring pore size after lyophilization is a relatively well-established and important method, and the errors introduced do not affect the comparison between groups [46]. The increase in mechanical performance enhances the durability of the hydrogel in SCI repair [47]. Considering the M-SpdHAMA hydrogel offers strong mechanical properties, suitable pore size for cell growth, appropriate porosity, and higher spermidine coupling, we selected M-SpdHAMA for its optimal characteristics. The healing period for mouse SCI is 28 days [37]. Through degradation and release curves, we found that as an implant, hydrogel exhibits a degradation rate that aligns with the healing process of mouse spinal cord, effectively releasing spermidine during degradation. Furthermore, by observing the hydrogel’s effective adhesion to the site of SCI throughout the reparative phase, we believe that hydrogel represents an excellent biomaterial implant suitable for spinal cord tissue applications.

The characterization of spermidine in hydrogels primarily includes its release kinetics within the gel and its stabilizing effect on the gel network. Through UV–visible spectrophotometry, experimental data confirmed reliable spermidine incorporation and time-dependent release from the hydrogel scaffold. Release profiling revealed an initial concentration-dependent increase in spermidine liberation from the hydrogel matrix. Previous studies have demonstrated that spermidine, a polyamine, possesses neuroprotective properties [16,18]. The neuron serves as the cornerstone of sensory and motor functions within the mammalian nervous system [48]. The hydrogel carrier AHAMA, designed by us, exhibits no toxic effects on neurons. This phenomenon may be attributed to the AHAMA hydrogel, which naturally incorporates ECM components, allowing it to mimic the ECM microenvironment. Post-degradation, there are no toxic metabolites causing neuronal damage or apoptosis. In vitro experiments showed that spermidine-loaded hydrogels exhibited no neurotoxicity to neurons and supported their normal growth. Furthermore, moderate spermidine loading within the hydrogel scaffold facilitated superior spinal cord regeneration.

Spermidine is a crucial metabolic polyamine in the human body, and fluctuations in spermidine levels have been implicated in driving neurodegenerative pathogenesis [18]. The underlying mechanism appears highly plausible to be the inhibitory effect of spermidine on chronic inflammation; however, the specific details of this mechanism have not yet been elucidated. Given that neuronal damage in SCI is largely dependent on excessive inflammatory responses at the site of injury, we focus on the role of spermidine-controlled release hydrogels in modulating inflammation in spinal cord trauma. Earlier investigations revealed that spermidine exerts notable immunomodulatory effects, primarily attenuating pro-inflammatory cytokine secretion and consequently mitigating inflammatory responses [49]. We have developed an SpdHAMA hydrogel for the treatment of SCI, capitalizing on spermidine’s pivotal role in regulating the nervous system. Through therapeutic interventions within an animal-based SCI pathophysiological model, we have observed that the SpdHAMA hydrogel facilitates neurons outgrowth at the injury site while inhibiting the proliferation of astrocytes. In addition, a substantial attenuation of M1 macrophage accumulation was observed at the injury site following SpdHAMA hydrogel application, which may be a potential mechanism by which SpdHAMA hydrogel exerts its pro-repair function. Further mechanistic exploration revealed that in vitro experiments demonstrated a significant inhibition of M1 macrophage polarization induced by SpdHAMA hydrogels. Western blot experiments indicated that SpdHAMA hydrogels can inhibit M1 macrophage polarization through the PTPN2–STAT1 axis. This suggests a potential mechanism underlying the pro-repair effects of SpdHAMA hydrogels. PTPN2 stands as a pivotal immunoregulatory tyrosine phosphatase [40]. This study, for the first time, elucidates the regulatory correlation between spermidine and PTPN2 within macrophages. Furthermore, the SpdHAMA hydrogel is adept at efficiently facilitating the controlled release of spermidine, suppressing M1 macrophage polarization, and promoting SCI repair. Therefore, this SpdHAMA hydrogel is anticipated to be employed in spinal decompression surgery, where it can be injected precisely onto severely damaged spinal cords, offering targeted repair, inflammation inhibition at the injury site, and facilitation of spinal cord motor function recovery. Although the SpdHAMA hydrogel demonstrates significant efficacy in promoting SCI repair in mice, it remains a considerable distance from clinical application. Further investigation involving the therapeutic use of spermidine hydrogel in primates is the next crucial step in our research agenda. Moreover, elucidating the regulatory mechanisms of spermidine on PTPN2 is also a critical therapeutic target.

In summary, the injectable SpdHAMA hydrogel prepared in this study effectively promotes SCI repair by up-regulating PTPN2 to inhibit M1 macrophage polarization. Importantly, the prepared hydrogel has shown promising in vivo efficacy in SCI repair, effectively adhering to the injury site, facilitating drug release, and filling the damaged area. This experimental research is poised to offer mechanistic insights into the future development of novel biomaterial implants for SCI repair. Further investigations into the application of injectable SpdHAMA hydrogel in primate SCI models and the specific molecular mechanisms underlying spermidine’s regulation of PTPN2 will be considerations for our next research steps.

## Conclusion

In this study, HA initially underwent a controlled reaction with sodium periodate, resulting in the formation of AHA. Simultaneously, AHA underwent a reaction with MA to AHAMA. Subsequently, the synthesis of SpdHAMA was achieved through a Schiff base conjugation reaction between spermidine and AHAMA. SpdHAMA hydrogels, prepared at various concentrations of spermidine, exhibited differences in morphology, porosity, swelling, and mechanical properties. All composite hydrogels demonstrated no cytotoxicity and were capable of inducing neuronal outgrowth. Preliminary in vivo experiments also provided initial evidence of the positive reparative effects of SpdHAMA hydrogel on SCI, with the ability to suppress M1 macrophage polarization, thus highlighting its potential application in spinal cord regeneration. Furthermore, this study elucidated the mechanism by which the spermidine hydrogel system inhibits M1 macrophage polarization through PTPN2. Overall, this research can provide experimental and theoretical foundations toward optimizing injectable biomaterial implants for translational use in SCI therapy.

## Ethical Approval

All animal studies were approved by the Ethical Committee of the Nanjing Medical University Animal Ethics Committee.

## Data Availability

The raw data supporting the conclusions of this manuscript will be made available by the authors, without undue reservation, to any qualified researcher.
