# Supplementary material for: Sustained-Release Spermidine Hydrogel Inhibits M1 Macrophage Polarization and Promotes Tissue Repair for Spinal Cord Injury Repair
Source: Biomater Res. 2025 Sep 11;29:0247. doi: 10.34133/bmr.0247 (PMC12423504; doi:10.34133/bmr.0247)
Supplement: Supplementary 1 — Figs. S1 to S4 [file bmr.0247.f1.docx]

**Supplementary data**

The following is the Supplementary data to this article:

Supplementary figure


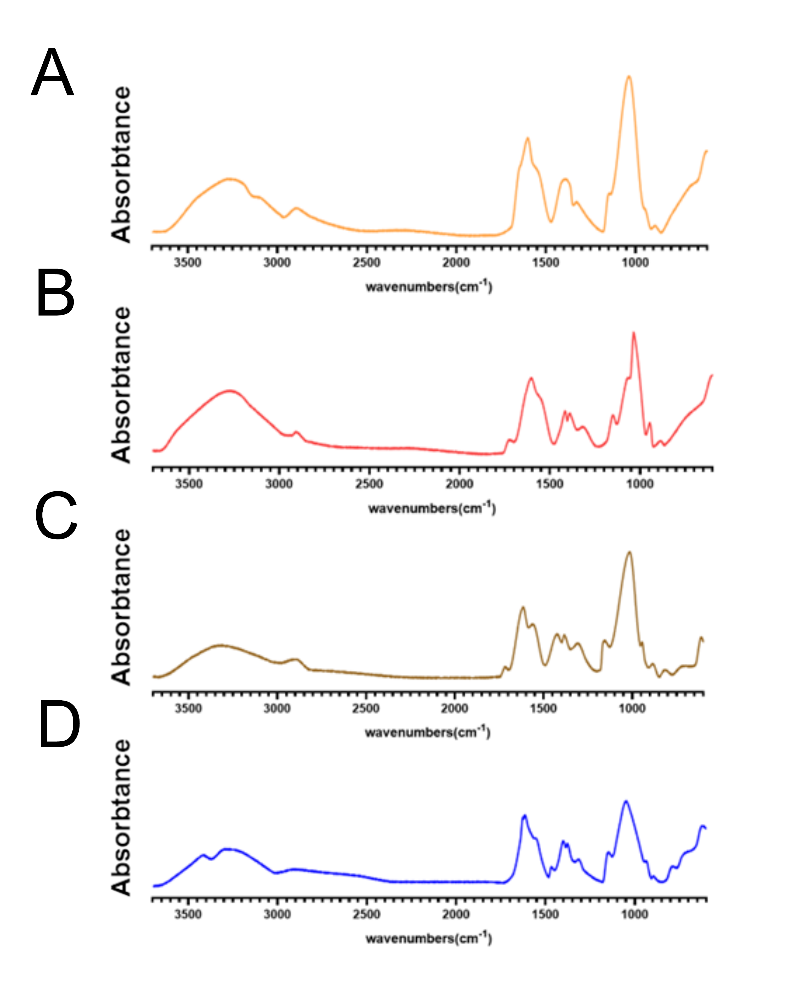


**Figure S1.** FTIR spectra of hyaluronic acid (HA) and its modified derivatives.

(A) FTIR spectrum of unmodified HA showing a broad absorption peak at 3200–3600 cm⁻¹ (–OH groups), carboxylate stretching vibrations at 1600 cm⁻¹ and 1400 cm⁻¹, and C–O–C and C–OH vibrations in the sugar ring at 1000–1200 cm⁻¹.

(B) FTIR spectrum of aldehyde-modified HA (AHA) with a characteristic C=O absorption peak at 1730 cm⁻¹, indicating the successful introduction of aldehyde groups and a slight reduction in hydroxyl peak intensity.

(C) FTIR spectrum of methacrylated HA (AHAMA) showing C=C bond stretching at 1630 cm⁻¹ and an ester C=O absorption peak at 1720 cm⁻¹, confirming successful methacrylation, along with further weakening of the hydroxyl peak.

(D) FTIR spectrum of spermidine-modified HA (SpdHAMA) showing a C=N stretching vibration at 1620 cm⁻¹ and N–H stretching vibrations at 3200–3500 cm⁻¹, indicating Schiff base formation. The C=O peak at 1730 cm⁻¹ significantly diminished, confirming the involvement of aldehyde groups in the grafting reaction with spermidine.


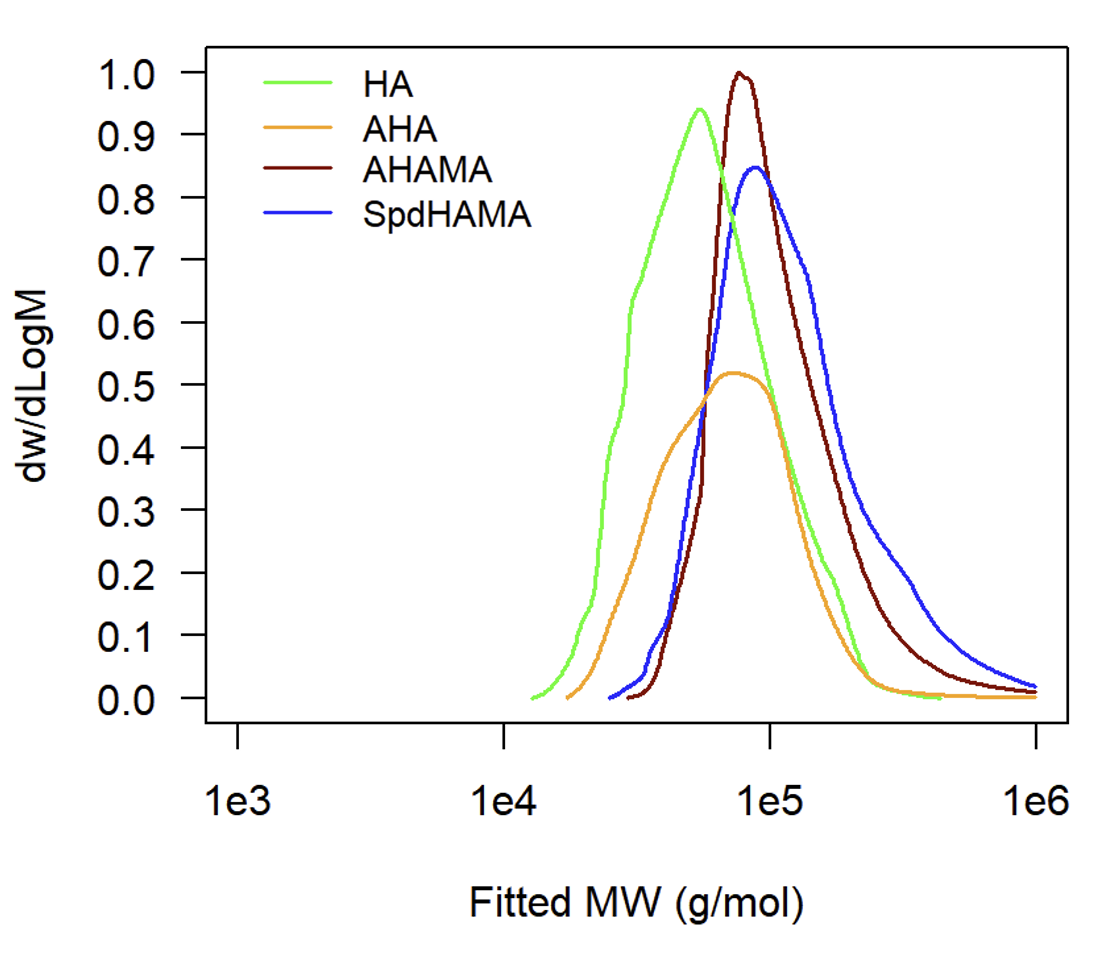


**Figure S2.** Fitted molecular weight distribution (dw/dlogM) of HA, AHA, AHAMA, and SpdHAMA measured by GPC


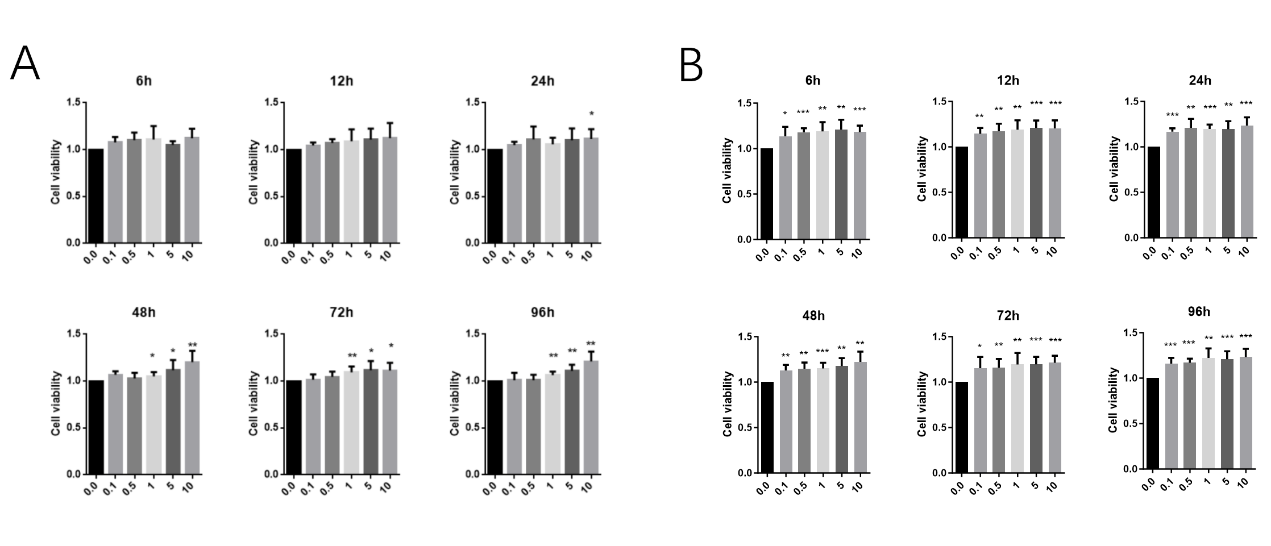


**Figure S3.** (A&B) The effect of the AHAMA hydrogel and the M-SpdHAMA hydrogel (0.1–10.0 mg/mL) on the viability of primary neurons (n = 6). *P < 0.05; **P < 0.01; ***P < 0.001.


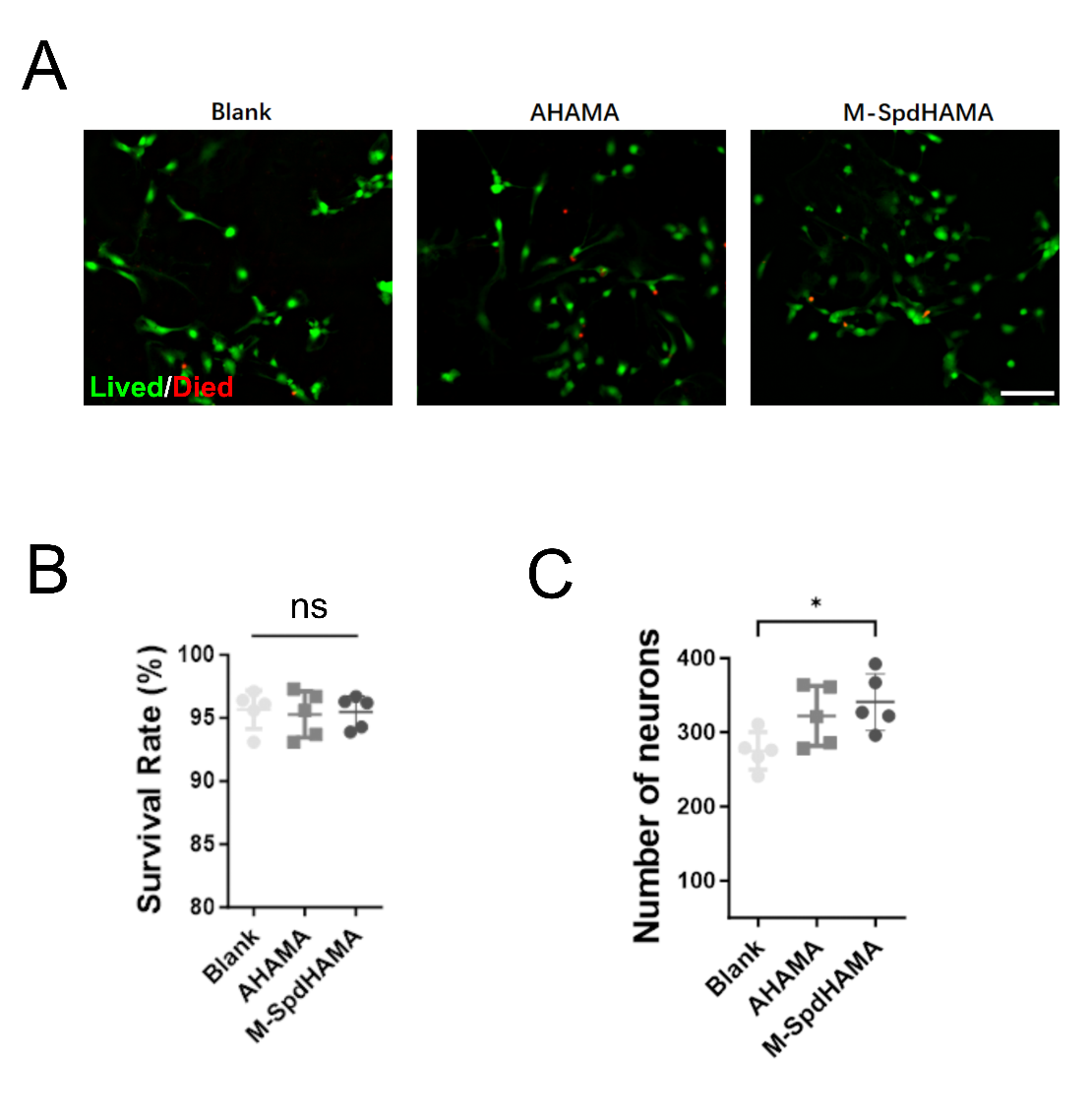


**Figure S4**. (A&B) Live/dead staining and quantification of co-cultured neurons among the Blank group, AHAMA group, and M-SpdHAMA group (n=5, Scale bar 100µm). (C) Quantitative analysis of the number of NeuN^+^, NF200^+^ and MAP2^+^ neurons in per field (n=5). *P < 0.05; **P < 0.01; ***P < 0.001; NS: No significant.
